# Fission yeast condensin contributes to interphase chromatin organization and prevents transcription-coupled DNA damage

**DOI:** 10.1186/s13059-020-02183-0

**Published:** 2020-11-05

**Authors:** Yasutaka Kakui, Christopher Barrington, David J. Barry, Tereza Gerguri, Xiao Fu, Paul A. Bates, Bhavin S. Khatri, Frank Uhlmann

**Affiliations:** 1 Chromosome Segregation Laboratory, The Francis Crick Institute, 1 Midland Road, London, NW1 1AT UK; 2 Waseda Institute for Advanced Study, Waseda University, 1-21-1, Nishiwaseda, Shinjuku-ku, Tokyo, 169-0051 Japan; 3 Bioinformatics & Biostatistics Science Technology Platform, The Francis Crick Institute, 1 Midland Road, London, NW1 1AT UK; 4 Advanced Light Microscopy Science Technology Platform, The Francis Crick Institute, 1 Midland Road, London, NW1 1AT UK; 5 Biomolecular Modelling Laboratory, The Francis Crick Institute, 1 Midland Road, London, NW1 1AT UK; 6 Department of Life Sciences, Imperial College London, Silwood Park Campus, Ascot, SL5 7PY UK

**Keywords:** Condensin, Chromosome architecture, Interphase chromatin, Transcription, DNA damage, Polymer physics, *S. pombe*

## Abstract

**Background:**

Structural maintenance of chromosomes (SMC) complexes are central organizers of chromatin architecture throughout the cell cycle. The SMC family member condensin is best known for establishing long-range chromatin interactions in mitosis. These compact chromatin and create mechanically stable chromosomes. How condensin contributes to chromatin organization in interphase is less well understood.

**Results:**

Here, we use efficient conditional depletion of fission yeast condensin to determine its contribution to interphase chromatin organization. We deplete condensin in G2-arrested cells to preempt confounding effects from cell cycle progression without condensin. Genome-wide chromatin interaction mapping, using Hi-C, reveals condensin-mediated chromatin interactions in interphase that are qualitatively similar to those observed in mitosis, but quantitatively far less prevalent. Despite their low abundance, chromatin mobility tracking shows that condensin markedly confines interphase chromatin movements. Without condensin, chromatin behaves as an unconstrained Rouse polymer with excluded volume, while condensin constrains its mobility. Unexpectedly, we find that condensin is required during interphase to prevent ongoing transcription from eliciting a DNA damage response.

**Conclusions:**

In addition to establishing mitotic chromosome architecture, condensin-mediated long-range chromatin interactions contribute to shaping chromatin organization in interphase. The resulting structure confines chromatin mobility and protects the genome from transcription-induced DNA damage. This adds to the important roles of condensin in maintaining chromosome stability.

## Background

Eukaryotic cells store their genetic information in the nucleus in the form of chromatin, a very long DNA-histone chain. Higher order chromatin organization plays essential roles in the regulation of gene expression and in the faithful inheritance of the genetic material. Structural maintenance of chromosomes (SMC) complexes are evolutionary conserved proteinaceous rings that control chromatin organization in organisms from bacteria to humans [1]. The SMC family member condensin is best known for its role in compacting chromatin in mitosis to form condensed and mechanically stable chromosomes [2]. Both fission yeast and budding yeast cells harbor a single type of condensin, whereas there are two distinct condensin complexes, condensin I and II, in higher eukaryotes. The condensin ring is formed by two SMC coiled-coil subunits and a kleisin subunit that bridges the two SMC subunits’ ATPase heads. Two accessory HEAT repeat subunits associate with the kleisin. Condensin I and II share the same SMC subunits but have different kleisin and HEAT subunits [2]. Both condensin complexes cooperatively contribute to chromosome condensation in mitosis [3, 4]. However, condensin I and II show different localization patterns during the cell cycle: condensin I is cytoplasmic during interphase and accumulates on chromatin only after nuclear envelope breakdown. Condensin II, on the other hand, associates with chromatin throughout the cell cycle [5]. Consistently, in addition to its mitotic role, condensin II has been attributed functions in interphase. These include chromosome territory formation [6, 7], as well as nurse cell polytene chromosome disassembly in the fruit fly [8]. The single fission yeast condensin complex shows nuclear accumulation in mitosis, a consequence of cyclin-dependent kinase phosphorylation of one of its SMC subunits, Cut3 [9]. In addition to its mitotic nuclear accumulation, fission yeast condensin is detectable in interphase nuclei at a lower level, where it has been reported to facilitate UV damage repair and the restart of stalled DNA replication forks [10]. Condensin also contributes to fission yeast chromosome territory maintenance in interphase [11].

Genome-wide chromatin contact formation has been investigated by high-throughput chromosome conformation capture, Hi-C. This revealed several levels of chromatin organization in interphase nuclei: A and B compartments, topologically associating domains (TADs), DNA loops, and stripes [12–16]. While A compartments correspond to transcriptionally active chromosome regions, B compartments encompass transcriptionally silent gene loci [12]. Chromatin contacts are more frequent within TADs than between TADs, which can favor interactions between gene regulatory elements [13]. Chromatin loops in turn stabilize promoter-enhancer interactions, while stripes are observed along actively transcribed gene bodies [14, 16]. Hi-C was used to determine condensin-dependent chromatin interactions during mitotic chromosome condensation in fission yeast [17, 18] and vertebrate cells [4]. This revealed the establishment of new long-range chromatin interactions by condensin in a distance range that is characteristic for the organism. These long-range interactions cause TADs to fuse in mitosis, while smaller interphase TADs are maintained by another SMC family member, cohesin [17–19]. Condensin is recruited to chromatin through an interaction with TFIIIC [20, 21] and other transcription factors [22], often to actively RNA polymerase II or III-transcribed genes [23, 24]. It is not yet clear how condensin’s role in interphase relates to its function in mitosis.

The dynamic behavior of chromatin, as a chain of nucleosomes, has been previously described. The mobility of individual nucleosomes was tracked [25], or a specific chromosomal locus traced by insertion of a Lac operator (LacO) array, bound by LacI conjugated to a fluorescent protein [17, 26–29]. The mean square displacement (MSD) in each case revealed subdiffusive chromatin motion. Chromatin mobility remains constant throughout interphase in human cells, where it is constrained by cohesin [25]. The mobility of budding yeast interphase chromatin approaches, but does not reach, that of a Rouse polymer, i.e., an ideal polymer chain [30]. Chromatin shows increased mobility following DNA damage, which is thought to facilitate efficient DNA repair [31–33]. Compared to interphase, fission yeast chromatin becomes highly confined in mitosis, dependent on condensin-mediated chromosome condensation [17]. Whether condensin also controls chromatin mobility during interphase is not yet known.

Here, we use an efficient combined transcriptional shut-off and protein degradation system [34] to study the roles of fission yeast condensin in spatial chromatin organization in the interphase nucleus. We deplete condensin while cells remain in a G2 interphase stage, to preempt indirect effects due to cell cycle progression in the absence of condensin. Hi-C revealed condensin’s contribution to interphase nuclear architecture, while chromatin mobility analysis uncovered that condensin constrains the movement of the chromatin chain. Strikingly, condensin depletion leads to the accumulation of transcription-induced DNA damage, exemplifying the importance of interphase chromatin organization by condensin for genome stability.

## Results

### Condensin maintains chromatin organization in interphase

**Fig. 1:**
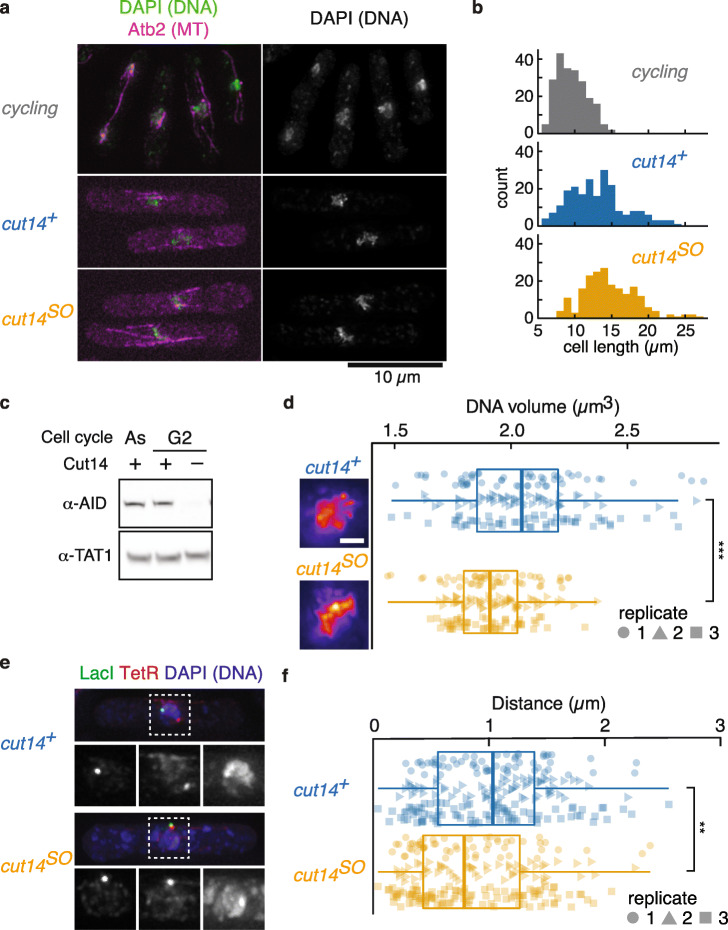
Condensin depletion causes loss of chromatin integrity in interphase. **a** Typical fluorescent images of exponentially growing (cycling) and G2-arrested cells in the presence (*cut14*^*+*^) or absence (*cut14*^*SO*^) of condensin. DNA, stained by DAPI, and mCherry-Atb2 (microtubules) are shown in green and magenta, respectively. Scale bar is 10 μm. **b** Distribution of cell lengths in cycling cells or following G2 arrest in *cut14*^*+*^ and *cut14*^*SO*^ strains (median cell lengths were as follows: cycling cells 9.7 μm, G2-arrested *cut14*^*+*^ cells 12.9 μm, and G2-arrested *cut14*^*SO*^ cells 14.3 μm). **c** Condensin depletion in G2-arrested cells was confirmed by western blotting. As, asynchronous cell culture; G2, cells arrested in G2 phase by 1NM-PP1. Cut14-AID was detected by α-AID antibody. α-TAT1 served as a loading control. **d** DNA volume quantified by DAPI staining. Boxplot of DNA volume in *cut14*^*+*^ control cells and following Cut14 depletion (*cut14*^*SO*^). Data from three biological replicates, identified by their distinct shapes, is compiled in one boxplot. ***p = 7.6 × 10^−8^. Pseudocolor images of DAPI-stained DNA in *cut14*^*+*^ and *cut14*^*SO*^ are shown as reference. Scale bar is 1 μm. **e** Typical images of chromosomal distance, visualized by LacI-GFP at the *lys1* locus (green, bottom left) and TetR-tdTomato at 1.95 Mb on chromosome I (red, bottom middle) in *cut14*^*+*^ and *cut14*^*SO*^. DNA stained by DAPI is shown in blue (bottom right). **f** Chromosomal distances in *cut14*^*+*^ and *cut14*^*SO*^ cells are displayed as in **d**. **p = 0.0012

To investigate the contribution of fission yeast condensin to chromatin organization in interphase, we combined a G2 phase cell cycle arrest with condensin depletion. First, cells were arrested in G2 by chemical inhibition of the ATP analogue-sensitive cyclin-dependent kinase (Cdk), *cdc2-as-M17* [35], using the ATP analogue 1NM-PP1. Before treatment, roughly 10% of cells showed mitotic markers (spindles or bi-nucleation). No such cells were detected following arrest. Fission yeast cells grow beyond their normal length during an arrest in G2, further confirming the arrest (Fig. 1a, b). The condensin SMC subunit Cut14 (known as Smc2 in other organisms) was now depleted by promoter repression due to thiamine addition and activation of its auxin-inducible degron by addition of the auxin indole-3-acetic acid (IAA) [36]. This treatment will hereafter be referred to as Cut14 shut-off (*cut14*^*SO*^), while control cells were of the same genotype but omitted thiamine and auxin addition (*cut14*^+^, see the “Methods” section for details) [34]. Efficient Cut14 depletion under these conditions was confirmed by western blotting (Fig. 1c; Additional File 1: Fig. S1).

To evaluate condensin’s role in interphase chromatin organization, we at first quantified the occupied DNA volume following 4′,6-diamidino-2-phenylindole (DAPI) staining. To accurately capture the volume, we acquired serial z-stacks (see the “Methods” section for details). Unexpectedly, the DNA volume in the absence of condensin was smaller when compared to control cells (Fig. 1d). The median occupied DNA volume in *cut14*^+^ control cells was 2.05 μm^3^, while that in *cut14*^*SO*^ cells was reduced to 1.91 μm^3^ (*p* = 7.6 × 10^−8^). As nuclear volume scales with cell size [37], we wondered whether cell size and therefore nuclear volume differences might account for the DNA volume effect. However, *cut14*^*SO*^ cells were equally, if not more, elongated compared to *cut14*^*+*^ cells at the time of imaging (Fig. 1b). Nuclear volume in *cut14*^*SO*^ cells was comparable to that in *cut14*^*+*^ cells, and the nucleus to cytoplasm (N/C) ratio was constant between our experimental conditions (Additional File 1: Fig. S2). These results suggest that the DNA volume reduction is not an indirect effect of nuclear size differences, but a consequence of condensin loss.

If DNA volume in interphase is indeed reduced in the absence of condensin, the linear distance between two chromosomal loci along a chromosome arm is also expected to be smaller. We therefore measured the distance between two chromosomal loci, separated by 1.8 Mb on the chromosome I left arm, visualized by LacO/LacI-GFP and TetO/TetR-tdTomato, respectively [17, 28]. Consistent with a DNA volume reduction, the two loci came closer in the absence of condensin (Fig. 1e, f). The median distance of 1.03 μm in G2-arrested control cells significantly shortened to 0.79 μm following condensin shut-off (*p* = 0.0012). To confirm this unexpected result, we repeated the experiment using a second auxin, 1-naphthaleneacetic acid (NAA), to deplete Cut14. This resulted in a similar shortening of the chromosome I arm distance from 1.01 to 0.80 μm (Additional File 1: Fig. S3). We conclude that condensin is required to maintain interphase chromosome volume and chromosome arm distances.

In mitosis, condensin acts to shorten the average distance between the same two chromosomal loci to 0.65 μm [17, 28], distinctly shorter than during interphase either with or without condensin. Taken together, this suggests that condensin fulfills a dual role in maintaining chromosome volume homeostasis. In interphase, a relatively low level of condensin maintains a chromosome volume that is greater than that of chromosomes lacking condensin. In mitosis, increased condensin levels achieve chromosome compaction. We will consider reasons for this initially counterintuitive behavior in the “Discussion” section.

### Chromatin domain architecture in the interphase nucleus

**Fig. 2:**
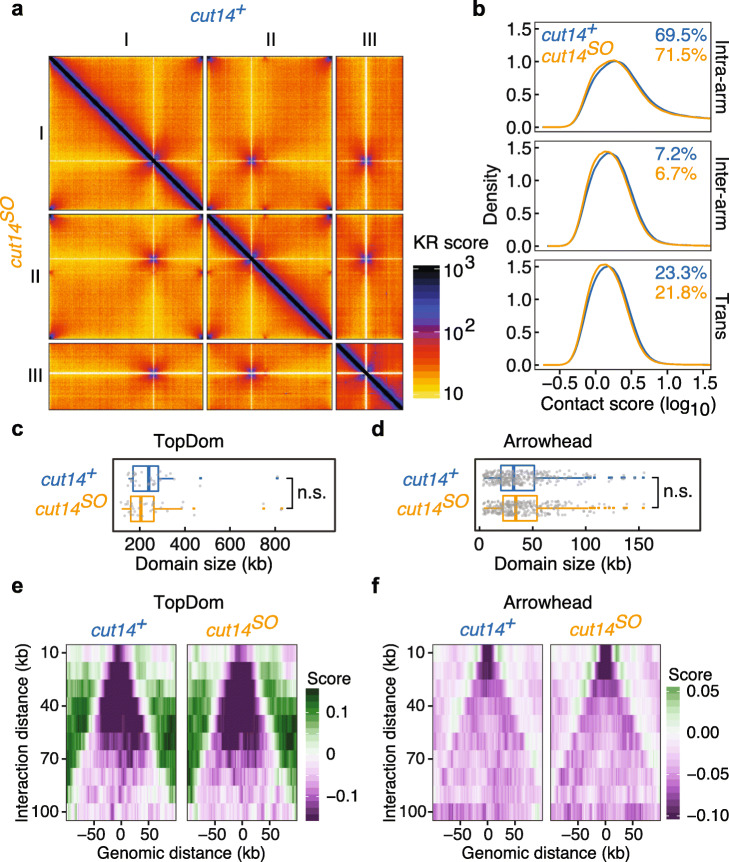
Spatial chromatin organization determined by Hi-C in the interphase nucleus. **a** Normalized Hi-C contact maps in *cut14*^*+*^ (top right) and *cut14*^*SO*^ (bottom left) conditions. **b** Distributions of chromatin interactions, categorized into intra-arm, inter-arm, and inter-chromosome (trans) interactions in *cut14*^*+*^ and *cut14*^*SO*^ cells. **c**, **d** Distribution of domain sizes determined by arrowhead (**c**) and TopDom (**d**) in *cut14*^*+*^ and *cut14*^*SO*^ cells. Dots indicate individual domain sizes. **e**, **f** Insulation scores at the boundaries, determined using the arrowhead (**e**) and TopDom (**f**) algorithms

To investigate how condensin maintains spatial chromatin organization in interphase, we performed Hi-C in G2-arrested cells, containing or depleted of condensin (*cut14*^*+*^ and *cut14*^*SO*^). We analyzed the detected interactions in bins of 2 kb resolution. At first sight, Hi-C maps showed equivalent configurations of chromatin contacts in *cut14*^*+*^ and *cut14*^*SO*^ cells (Fig. 2a).

We then categorized chromatin interactions into those within chromosome arms (intra-arm), those between the two arms of the same chromosome (inter-arm), and those between different chromosomes (trans). While the frequency distributions within each class were only marginally affected by the absence of condensin, the overall proportion of intra-arm interactions significantly increased in the absence of condensin, at the expense of inter-arm and trans interactions (Fig. 2b). Intra-arm contacts account for 69.5% of interactions in control cells, but increased to 71.5% following condensin inactivation. The reduced DNA volume following condensin depletion should favor intrachromosomal interactions and is consistent with this observation. On the other hand, the chromatin interaction data did not recapitulate the microscopically observed intermixing of chromosome territories in the absence of condensin [11]. The different size regime of microscopic observations in the micrometer range, from the nanometer-scale interactions captured by Hi-C, might be a reason for this difference.

We next analyzed the contribution of condensin to interphase TAD formation. Such a role might be expected from previous observations that fission yeast condensin and vertebrate condensin II contribute to cytologically observed chromosome territory formation [6, 7, 11]. We performed chromosome domain calling using both the arrowhead [14] and TopDom [38] algorithms. The arrowhead analysis identified 32 and 36 domains in *cut14*^*+*^ and *cut14*^*SO*^ cells, with a median size of 240 kb and 205 kb, respectively (Fig. 2c), suggesting only a minor condensin contribution to domain architecture. The more sensitive TopDom analysis identified 290 smaller domains with a median size of 32 kb in control cells and 275 domains with a median size of 34 kb in *cut14*^*SO*^ cells (Fig. 2d). The small differences in either case were not statistically significant (*p* = 0.27 and *p* = 0.09, respectively). Additionally, we examined the insulation scores at domain boundaries determined by both methods (Fig. 2e, f). This revealed that condensin shut-off did not affect insulation at the domain boundaries. We conclude that condensin only weakly impacts on the interphase chromatin domain architecture in fission yeast. The domain sizes identified in our TopDom analysis are consistent with those that have previously been ascribed to the cohesin complex [19, 39]. These results therefore suggest that the fission yeast chromatin domain architecture in interphase is predominantly shaped by cohesin-mediated contacts.

### Condensin establishes long-range chromatin interactions in interphase

**Fig. 3:**
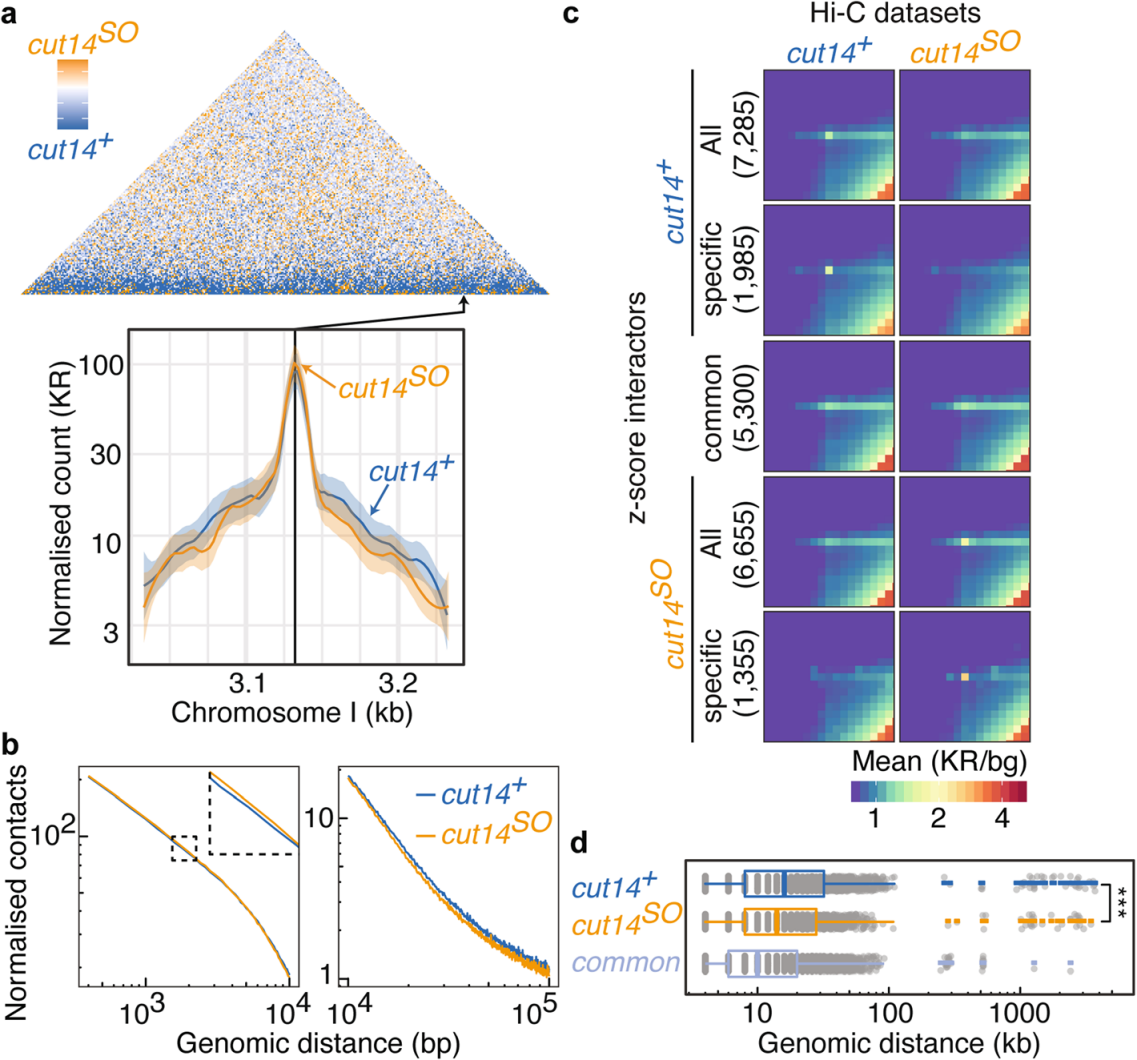
Hi-C reveals condensin-dependent chromatin interactions. **a** Hi-C difference map of the chromosome I left arm comparing *cut14*^*+*^ and *cut14*^*SO*^ cells, as well as virtual 4C plots around the indicated viewpoint. Solid lines and shaded areas represent Loess-smoothed Hi-C contacts and 95% confidence intervals, respectively. **b** Contact probability as a function of genomic distance along the chromosome I left arm in *cut14*^*+*^ and *cut14*^*SO*^ cells. **c** Aggregate Hi-C matrices of high *z*-score interactions in the indicated conditions. All, all interactions identified in either *cut14*^*+*^ or *cut14*^*SO*^ cells; specific, interactions seen only in one of the two conditions; common, interactions identified in both conditions. Numbers of interactions are shown in brackets. **d** Genomic distances of high *z*-score interactions plotted as boxplots. ***p = 0.0002

To visualize condensin-mediated chromatin interactions in interphase, we prepared a Hi-C difference map by subtracting normalized Hi-C contact frequencies in control cells from the ones in *cut14*^*SO*^ cells (Fig. 3a and Additional File 1: Fig. S4a). This revealed a band of interactions parallel to the diagonal that were reduced in the absence of condensin. In contrast, small regions on the diagonal showed increased interactions in *cut14*^*SO*^ cells. A virtual 4C plot using a locus on the chromosome I right arm as a viewpoint exemplifies a substantial increase of local chromatin contacts as well as a reduction of long-range interactions in *cut14*^*SO*^ cells compared to the *cut14*^*+*^ control (Fig. 3a).

To better understand the distance regime in which condensin engages in interphase interactions, we plotted the interaction frequency of all loci as a function of chromosomal distance. This revealed that condensin promotes increased interactions between around 100 kb and 1 Mb, while shorter chromatin contacts were more pronounced in the absence of condensin (Fig. 3b). The size regime of condensin-mediated chromatin contacts is thus similar in interphase, when compared to chromosome condensation in mitosis [17], although the magnitude of condensin-mediated interactions is much less prominent in interphase.

A contribution of condensin to long-range chromatin contacts in interphase also became apparent when we recorded the distribution of all interaction distances that emanate from a given genomic locus. When plotted as a function of chromosome coordinate, the medians of these distributions were shorter in *cut14*^*SO*^ cells along all three fission yeast chromosomes (Additional File 1: Fig. S4b). The genome-wide median interaction distance of 38 kb shortened to 36 kb following condensin depletion (*p* = 3.7 × 10^−54^).

The *z*-score is the number of standard deviations that the interaction frequency between any two loci lies above or below the mean interaction frequency at this given distance. A *z*-score Hi-C map revealed numerous strong chromatin contacts at different genomic distances (Additional File 1: Fig. S4c). Interactions with a high *z*-score are likely biologically relevant chromatin loops, so we analyzed all those interactions with a *z*-score of greater than 2. Using this criterion, we identified a total of 7285 and 6655 interactions in *cut14*^*+*^ and *cut14*^*SO*^ cells. Five thousand three hundred of these were common to both conditions whereas 1985 and 1355 interactions were specific to *cut14*^*+*^ and *cut14*^*SO*^ cells, respectively. Aggregate Hi-C matrices illustrate that the *z*-score analysis indeed identified chromatin loops specific to both conditions (Fig. 3c). Notably, the interaction distances covered by these high *z*-score interactions were significantly larger in the presence of condensin (Fig. 3d). This corroborates the idea that condensin establishes long-range chromatin loops. In contrast, interactions that were seen in both *cut14*^*+*^ or *cut14*^*SO*^ cells, which are therefore condensin-independent, were markedly shorter than interactions specific to either of the conditions. The aggregation matrix analysis further revealed a point-shaped characteristic of condensin-specific interactions, suggesting that condensin engages in discrete loop interactions (Fig. 3c). Interactions common to both conditions showed a stripe-like appearance, indicative of a condensin-independent interaction mechanism that engages broader chromosome regions in the fission yeast interphase nucleus.

### Condensin reduces chromatin mobility in interphase

**Fig. 4:**
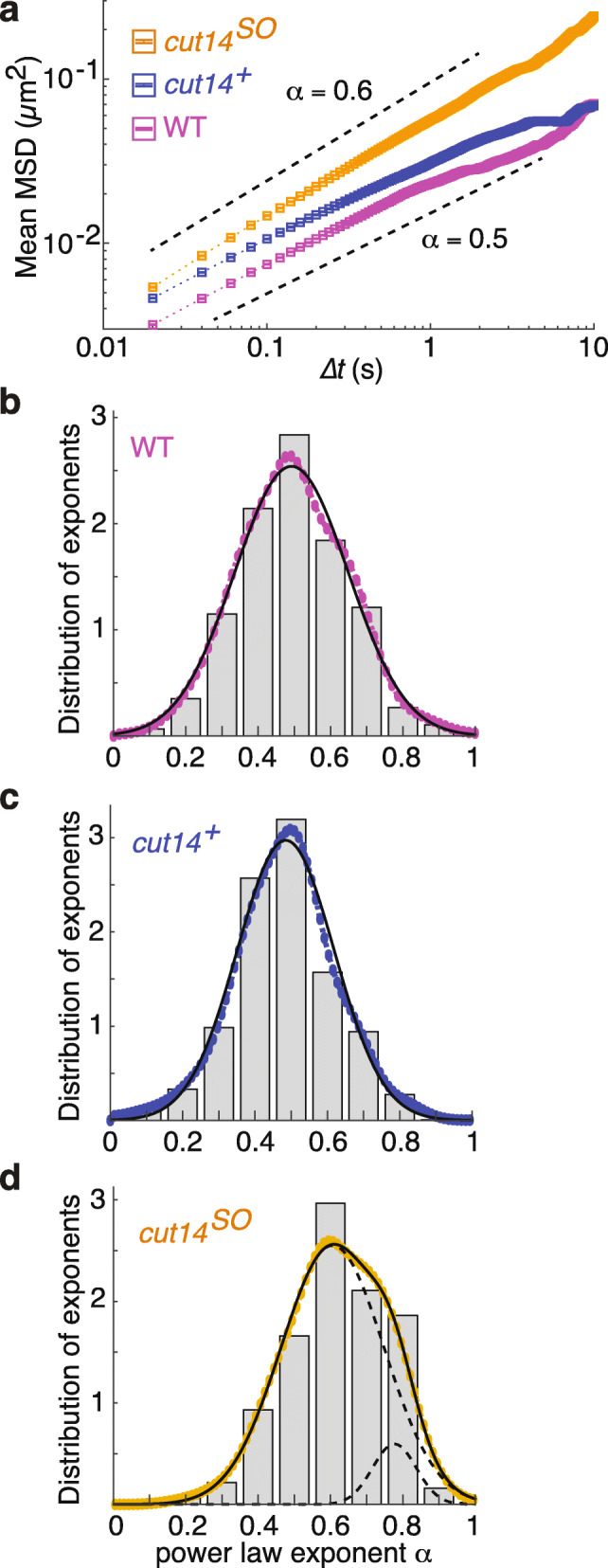
Condensin confines chromatin mobility in interphase. **a** MSD of chromatin mobility in wild type (WT), control (*cut14*^*+*^), and following condensin depletion (*cut14*^*SO*^). Weighted mean (dotted line) and standard error (square boxes) are shown. Black dotted lines above and below mean MSD are the slopes corresponding to 0.6 and 0.5, respectively. **b**–**d** Histogram (gray bars) and kernel density estimate (colored circles) of probability density of individual MSD exponents in G2-arrested WT (**b**), *cut14*^*+*^ (**c**), and *cut14*^*SO*^ (**d**) cells. Solid lines are single Gaussian fit in case of WT and *cut14*^*+*^ and a double Gaussian fit for *cut14*^*SO*^ cells. The dotted lines show the two component Gaussians (*n* > 300)

Condensin confines chromatin mobility when fission yeast chromosomes condense in mitosis [17]. We therefore wondered whether condensin also restricts chromatin movement in interphase. We monitored chromatin mobility by high-speed confocal microscopy in G2-arrested cells in the presence or absence of condensin. To avoid possible impact from chromatin tethering to the nuclear envelope [40], we recorded the dynamics of LacO arrays inserted in the middle of the long left arm of chromosome I (1.95 Mb from the left telomere and 1.8 Mb from the centromere). To monitor the rapid chromatin motion, we captured single focal plane images in 20-ms intervals. As a further control in these recordings, in addition to the experimental strain (*cut14*^*+*^ or *cut14*^*SO*^), we also included a wild-type strain without any modification to the *cut14* locus (referred to as WT). Mean squared displacement (MSD) of the LacO arrays was then plotted over time. Mean MSD plots revealed an increased mobility of the LacO-marked chromatin locus in the absence of condensin (Fig. 4a). The mean MSD exponents α under these conditions were 0.52 and 0.49 for WT and *cut14*^*+*^ cells, respectively, while α increased to 0.60 in *cut14*^*SO*^ cells. These results indicate that fission yeast chromatin moves in a subdiffusive manner in interphase, as observed in other eukaryotes [26, 27, 41, 42], and they demonstrate that condensin limits this mobility.

To explore additional features of chromatin mobility, we examined the individual MSD trajectories (Additional File 1: Fig. S5a-c). MSD linearly increased for the first 0.5 s, then the noise level increased, probably due to nuclear or chromosome movements additional to the local chromatin chain mobility. We therefore used the time interval from 0 to 0.5 s for the following analyses. The MSD exponents of individual trajectories were determined by regression, then depicted using a binned histogram (Fig. 4b–d). We used kernel density smoothing using a Gaussian kernel to estimate the mean MSD exponent in each condition. A single Gaussian provided a good fit for the exponent distributions under both the WT and *cut14*^*+*^ conditions. The mean MSD exponent was 0.49 in both cases, consistent with our above analysis. In contrast, a single Gaussian did not provide a good fit for the exponent distribution under *cut14*^*SO*^ conditions. We therefore applied two Gaussians, which provided an improved fit (Fig. 4d and Additional File 1: Fig. S5d). The main Gaussian distribution encompasses 91% of the population and has a mean exponent of 0.61, again consistent with what we deduced from our initial analysis (Fig. 4a). However, our analysis suggests that a smaller second population exists that includes approximately 9% of the population and displays a greater mean MSD exponent of 0.78. These results suggest that condensin not only confines chromatin mobility, but also prevents the occurrence of cells with exceptionally mobile chromatin. We will discuss the possible origin of such highly mobile chromatin in the absence of condensin, below.

All the above analyses were carried out using cells arrested in the G2 phase of the cell cycle by chemical Cdc2 kinase inhibition. To establish whether the cell cycle arrest affected chromatin mobility, we also recorded chromatin tracks in asynchronously growing fission yeast cells, most of which are in the G2 cell cycle phase. We used both WT and *cut14*^*+*^ cells for this analysis and again plotted histograms of the exponents obtained from individual MSD tracks (Additional File 1: Fig. S6). Single Gaussian fitting provided good descriptions of these distributions with mean MSD exponents of 0.49 and 0.48 in WT and *cut14*^*+*^ cells, respectively. These values are very close to those observed in G2-arrested cells and suggest that chromatin mobility was not affected by Cdc2 kinase inhibition or the consequent G2 arrest. Unfortunately, we could not determine the role of condensin in asynchronously growing cells, as we were unable to find fast enough depletion conditions. Together, our results suggest that an MSD exponent of around 0.5 describes the nature of chromatin behavior in fission yeast interphase cells and that condensin is constraining this exponent.

### Following condensin depletion, chromatin behaves like a Rouse polymer with excluded volume

The MSD exponent of fission yeast interphase chromatin is roughly 0.5, consistent with previous observations in budding yeast [27, 42] and mouse pro-B cells [41]. It is well known that a Rouse polymer, i.e., an ideal polymer chain of beads subjected to Brownian motion and connected by harmonic springs, displays an MSD exponent of 0.5 [43]. Because chromatin is a chain-like polymer of nucleosomes, this broad agreement means that a Rouse polymer has been widely utilized to model chromatin dynamics [30]. On the other hand, our experiments show that condensin depletion leads chromatin to display a larger MSD exponent of approximately 0.6, more than what is reached by a Rouse polymer. How can we explain the larger MSD exponent following condensin depletion by the theory of polymer physics?

An important aspect of an ideal polymer chain is that it is free to cross itself. Real polymer chains differ from ideal polymer chains in that they occupy a volume. This volume is inaccessible to other parts of the chain, and it restricts the ability of the chain to cross itself [44]. Consequently, the chain is forced to explore new areas, which increases the MSD exponent. In the case of chromatin, nucleosomes occupy a volume and they cannot pass through each other. Therefore, a Rouse polymer chain with excluded volume is a more realistic model for a chromatin chain in vivo. In a “good” solvent, where attractive chain-chain interactions are not dominant, we can use theory to predict an exponent α ≈ 0.54 for a Rouse polymer with excluded volume (see the “Methods” section). This approaches the observed MSD exponent in the absence of condensin, though additional factors must contribute to arrive at the measured exponent of 0.6.

**Fig. 5:**
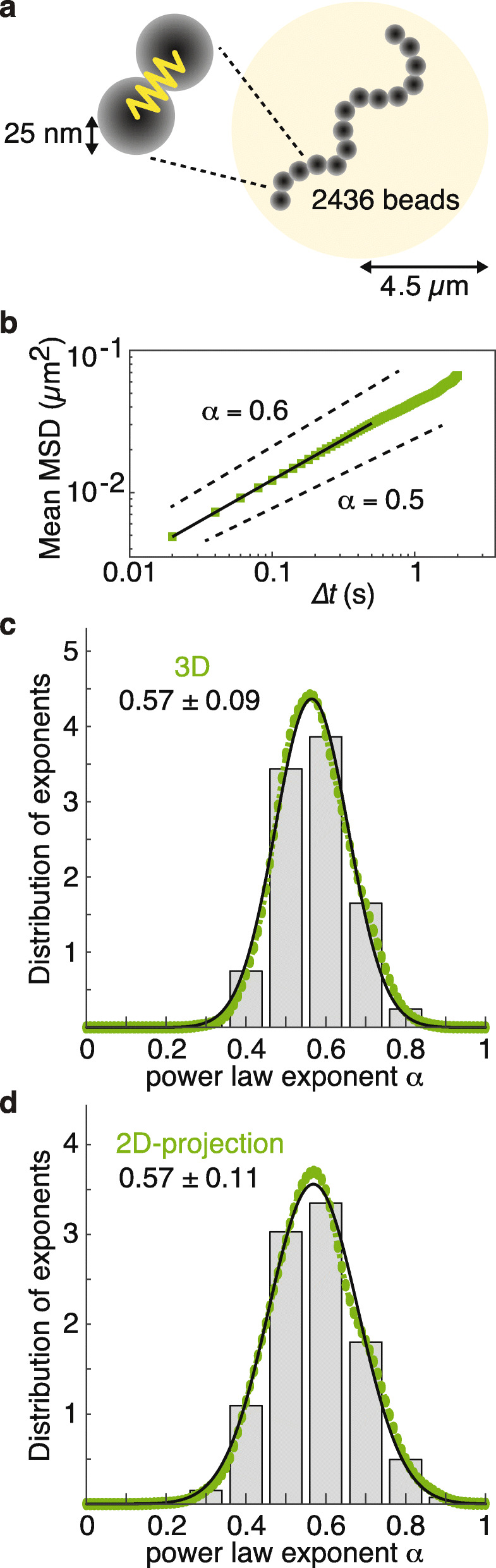
Simulation of a coarse-grained Rouse polymer with excluded volume. **a** Schematic illustration of a coarse-grained Rouse polymer with excluded volume. **b** Mean MSD of a Rouse polymer with excluded volume (green) including a linear fit with slope α = 0.57 (black line). Dotted lines show the slopes corresponding to 0.5 and 0.6. **c**, **d** Histogram of MSD exponents from the model, displaying a single Gaussian fit (solid lines) and kernel density estimate of probability density of individual MSD exponents (colored circles) in 3D (**c**) and 2D projection (**d**). The means and standard deviations are indicated

As an alternative approach to estimate the MSD exponent of a Rouse polymer with excluded volume, we used a computational beads-and-spring polymer model to recapitulate chromatin dynamics. We modeled 2436 beads, each representing 10 nucleosomes, approximating the length of the fission yeast chromosome I left arm. The beads have a 25-nm radius and move according to Brownian motion within the overall confines of a 4.5-μm-radius sphere, allowing largely unconstrained movement (see the “Methods” section for details). We find that the mean MSD exponent of beads in this simulation is 0.57 (Fig. 5a, b), which is slightly larger than we theoretically predicted and close to what we observed in vivo following condensin depletion.

If attractive chain-chain interactions are added to a Rouse polymer with excluded volume, this reduces its mobility and resultant MSD exponent. One way of interpreting condensin’s role is therefore that it provides polymer chain-chain attraction. It appears to do so to an extent that brings the observed MSD exponent of a Rouse polymer with excluded volume effects back to 0.5. While this appears numerically similar to an ideal polymer chain, it is physically quite distinct.

The bead tracking in the polymer simulations was performed in three dimensions, while our chromatin mobility measurements in live cells were restricted to two dimensions. To clarify how this difference might affect the MSD exponent, we modified our computational analysis. We projected the 3D positions of simulated beads onto a random 2D plane, mimicking our in vivo image acquisition, and tracked the projected bead positions. We then plotted the MSD exponent distributions based on both 3D tracking and 2D projections side by side and fitted single Gaussian kernel density estimates (Fig. 5c, d and Additional File 1: Fig. S7). The mean MSD exponents were 0.57 in both cases, suggesting that the 2D projection does not alter the mean MSD exponent. However, we note that the MSD exponent distribution became somewhat broader as a result of the 2D projection. These results indicate that chromatin behavior in interphase, once condensin is removed, is roughly consistent with that of a beads-and-spring polymer with excluded volume. The greater observed in vivo MSD exponent of 0.6, compared to that predicted theoretically (0.54) or empirically (0.57), could be due to dynamic measurement errors, which are known to induce an overestimation of exponents [45]. Alternatively, additional chromosomal activities could impart motion, e.g., chromatin remodelers or other aspects of gene transcription [46].

### Condensin prevents spontaneous transcription-induced DNA damage

A small fraction of MSD trajectories following condensin depletion displayed an unusually high exponent, and we wondered what the cause of this highly mobile chromatin was. The MSD exponent of 0.78 observed for approximately 10% of trajectories following condensin depletion is close to an exponent $\alpha =\frac{3}{4}$ for a semi-flexible polymer [47, 48]. A semi-flexible polymer is locally stiffer and has a longer persistence length. This is reminiscent of the increased chromatin MSD exponent seen in response to double-stranded DNA breaks in budding yeast, a possible explanation for which is thought to be chromatin stiffening [32, 46, 49–51]. We therefore wondered whether DNA damage was the reason for a fraction of cells with increased chromatin mobility following condensin depletion.

**Fig. 6:**
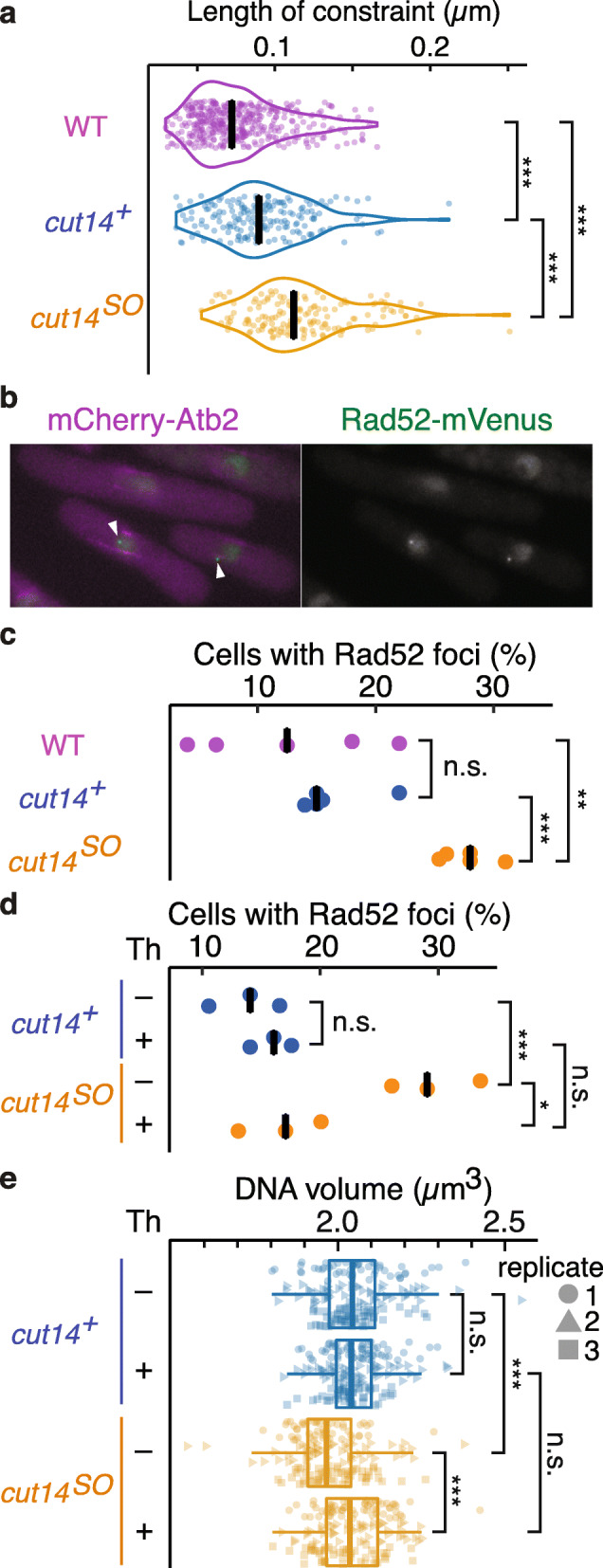
Transcription-induced DNA damage in the absence of condensin. **a** Violin plots of lengths of constraint (*L*_c_) in the indicated strains. ***p = 7.6 × 10^−12^ (WT vs *cut14*^*+*^), *p* = 1.3 × 10^−51^ (WT vs *cut14*^*SO*^), *p* = 2.6 × 10^−16^ (*cut14*^*+*^ vs *cut14*^*SO*^) (*n* > 300). **b** Image of cells with Rad52 foci in *cut14*^*SO*^. White arrowheads point to Rad52 foci. **c** Fraction of cells in the indicated conditions with Rad52 foci (five biological replicates, *n* ≥ 74); black bars show the median. n.s., no statistical significance; **p = 0.009, ***p = 0.0003. **d** Fraction of *cut14*^*+*^ and *cut14*^*SO*^ cells with Rad52 foci following control or thiolutin (Th) treatment (three biological replicates, *n* = 200). Black bars show the median. n.s., no statistical significance; *p = 0.013, ***p = 0.005. **e** DNA volumes were quantified by DAPI staining as in Fig. 1d. Boxplots show DNA volumes in *cut14*^*+*^ and *cut14*^*SO*^ cells with or without thiolutin (Th) treatment. Data from three biological replicates (*n* > 50), identified by their distinct shapes, is compiled into one boxplot. n.s., no statistical significance; ***p < 2.53 × 10^−8^

Previous studies on chromatin behavior in response to DNA damage have highlighted an increased length of constraint, *L*_c_, as a consequence of DSB formation [31–33]. We therefore first calculated *L*_c_ from our chromatin trajectories in the presence and absence of condensin. *L*_c_ is the standard deviation of the locus position to the average position over the recorded time. It is an estimate for the radius of the area that the locus explored. In budding yeast, *L*_c_ was reported as 0.13 μm, which increased to 0.23 μm after DSB formation [33]. *L*_c_ in our recordings in fission yeast was 0.076 μm in WT and 0.093 μm in *cut14*^*+*^ cells, which increased to 0.114 μm in *cut14*^*SO*^ cells (Fig. 6a). Thus, as observed in response to DNA damage in budding yeast, both the MSD exponent and the length of constraint increase in interphase fission yeast cells depleted of condensin.

To examine if the above effects are due to DNA damage, we monitored the formation of Rad52 foci, which is a mark of DSB formation [52] (Fig. 6b). We again arrested cells in G2, after which we depleted condensin. Rad52 foci formation was visualized using a Rad52-mVenus fusion. The fraction of cells with Rad52 foci substantially increased following condensin depletion (Fig. 6c). Twenty-eight percent of *cut14*^*SO*^ cells displayed discernible foci, while this fraction was around 10% of WT or *cut14*^*+*^ cells. These results suggest that condensin is required in interphase to prevent the accumulation of spontaneous DNA breaks. The DNA damage in turn is a plausible cause for increased chromatin mobility in condensin-depleted cells.

What might cause DNA breaks in the absence of condensin? Condensin promotes repair of exogenous DNA lesions caused by UV irradiation [10]. It could therefore be that condensin also helps to repair spontaneous endogenous DNA damage, e.g., caused by reactive oxygen species. Alternatively, active nuclear processes could produce DNA damage if the interphase chromatin architecture is compromised by the absence of condensin. To differentiate between these possibilities, we inactivated a prevalent nuclear process, gene transcription, using the transcription inhibitor thiolutin. Addition of thiolutin at the time of condensin depletion did not affect the condensin depletion efficiency (Additional File 1: Fig. S8a). However, it substantially suppressed the formation of Rad52 foci (Fig. 6d). This suggests that a transcription-coupled process inflicts genome damage, but that condensin-mediated chromatin architecture protects against this damage.

An alternative explanation for the transcription dependence of Rad52 foci is that transcription is not a cause of the damage but that transcription is required for Rad52 foci formation as part of the DNA damage response. To test this, we treated cells with the irradiation mimetic phleomycin. This led to Rad52 foci formation irrespective of whether transcription was active or was inhibited by thiolutin (Additional File 1: Fig. S8b). We conclude that transcription is not required for DNA damage signaling at least up to the point of Rad52 foci formation. Rather, transcription might be an actual cause of damage formation in the absence of condensin.

Lastly, we tested whether the DNA volume compaction that we observed in the absence of condensin is a consequence of DNA damage. Thiolutin addition markedly suppressed the DNA volume reduction following condensin depletion (Fig. 6e). This suggests that a transcription-coupled process inflicts genome damage, which results in a reduction of DNA volume, but that condensin-mediated chromatin architecture protects against such damage.

## Discussion

In this study, we find that fission yeast condensin mediates long-range chromatin interactions and confines chromatin mobility in interphase, as it does in mitosis [17]. While quantitatively the effect in interphase is far less pronounced, it makes a crucial contribution to genome stability.

Fission yeast condensin shuttles between the cytoplasm and the nucleus. While the equilibrium is biased towards the cytoplasm for much of the cell cycle, condensin accumulates in the nucleus following mitotic Cdk phosphorylation of its Cut3 subunit on threonine 19 [9]. It could therefore be that fission yeast condensin retains a constant biochemical activity, except for its cell cycle-regulated subcellular localization. The quantitative increase in condensin-mediated long-range chromatin interactions in mitosis would then be the mere consequence of the rising nuclear condensin concentration at this time. On the other hand, Cdk phosphorylation of vertebrate condensin increases its in vitro DNA supercoiling activity and is a prerequisite for its ability to form chromosomes in vitro [53]. In budding yeast, the dynamic turnover of condensin on chromosomes is slowed down following mitotic Cdk phosphorylation [54]. We therefore cannot discount the possibility that fission yeast condensin is also cell cycle regulated in ways additional to its subcellular localization. In either case, our results suggest that condensin impacts on chromatin in a principally similar way in both interphase and mitosis. Future work will further explore how cell cycle-dependent modifications regulate condensin’s biochemical activities and in vivo behavior.

Given that condensin engages in long-range chromatin interaction in both interphase and mitosis, it came as a surprise to observe that the interphase chromosome volume decreases following condensin depletion. The opposite is found in mitosis when chromosomes increase in volume following condensin depletion [17, 28, 55]. We can imagine two scenarios to explain this conundrum. The first relates to the DNA damage that results from condensin depletion. Damaged DNA is organized in clusters [56], and this in turn might compact the DNA. If we think about chromosome volume from a polymer physics viewpoint, damaged DNA is divided into smaller segments and again these might occupy a smaller volume. We should keep in mind that G2 chromatin exists bound to its sister chromatid by cohesin, giving a ladder-like polymer with a certain persistence length. A DNA break could reduce the persistence length as a longitudinal link on one of the two axes is broken, allowing the resultant more flexible chain to adopt a more compact structure. Consistent with this interpretation, transcription inhibition suppressed DNA damage as well as DNA volume reduction following condensin depletion. A drawback of this explanation is that volume compaction following condensin depletion was observed in all cells, while DNA damage and Rad52 foci formation were evident in only a subset of cells.

We can consider an alternative possibility how condensin contributes to chromosome volume homeostasis by DNA looping. Condensin has been observed to actively extrude DNA loops under certain conditions in vitro [57]. If a similar phenomenon occurs in vivo, converting a linear polymer into short loops could lead to a condensin-dependent volume increase. Whether condensin indeed autonomously extrudes DNA loops in vivo remains uncertain. Condensin could alternatively form loops by sequential DNA capture, which then expand while condensin moves along transcription units driven by RNA polymerases as an extrinsic motor [20, 23, 58]. Irrespective of the underlying mechanism for loop formation, absence of looping following condensin depletion could result in the observed interphase volume contraction. At greater condensin concentrations in mitosis, loop clustering and maybe loop nesting will lead to chromosome compaction that condensin is known to achieve at this time. Further investigations into how condensin generates loops, complemented by computational and theoretical approaches, will be required to understand condensin’s role in chromatin organization.

Our experimental system provided a unique opportunity to determine the impact of condensin depletion in interphase, while avoiding confounding effects from cell cycle progression with compromised condensin function. This revealed that condensin is required to prevent the spontaneous generation of Rad52 DNA repair foci in the interphase nucleus. Consistent with a role of condensin in protecting against intrinsic DNA damage, *Arabidopsis* condensin II mutants display a DSB signature [59] and *smc2* knockdown in mouse ES cells induces γ-H2AX DNA repair foci [60]. Furthermore, the fission yeast *cnd2-1* mutation elicits a checkpoint-dependent mitotic entry delay even in the absence of exogenous damage [10]. But what might be the source of DNA damage? The observation that thiolutin treatment prevents damage foci formation suggests that it is a process that is coupled to transcription. Single-stranded DNA is exposed following fission yeast condensin inactivation [61], again dependent on active transcription [23]. Together with the observation of naturally occurring single-stranded DNA breaks at promotors of active genes and transcription-dependent promoter decompaction [62, 63], this highlights the challenge to genome stability that arises from DNA unwinding and topological strain associated with gene transcription. To understand how condensin conveys its protective effect, it will be important to map locations of preferential condensin binding in the interphase nucleus, as well as the locations of the fragile sites where DNA breaks in its absence.

## Conclusions

Condensin was discovered as a key protein component of mitotic chromosomes that bestows them with their characteristic shape and allows their successful segregation during cell divisions. We have begun to appreciate how this genome architect uses a fraction of its potency throughout the cell cycle to contribute to interphase chromatin architecture. Future work will explore how this protects the genome and makes gene expression possible without collateral damage to the DNA.

## Methods

### *Schizosaccharomyces pombe* strains and culture

All the strains used in this study are listed in Additional File 1: Table S1. PCR-based gene tagging [64] was used to construct the Rad52-mVenus strains, while the other strains were constructed by standard genetic manipulation [65].

All the strains were cultured in Edinburgh minimal medium (EMM) supplemented with adenine, leucine, uracil, lysine, histidine, and l-glutamate as a nitrogen source and 2% glucose for a carbon source. To deplete condensin in G2-arrested cells, cells were cultured with 1 μM 1NM-PP1 and 5 μg/ml thiamine for 90 min at 25 °C. Then, 0.5 mM of either indole-3-acetic acid (IAA) or 1-naphthaleneacetic acid (NAA) was added to the culture and incubated another 90 min at 25 °C. For the control cells arrested in G2 cell cycle stage, the equivalent amount of the respective solvent methanol or ethanol was added to the culture after the inhibition of Cdk by 1 μM 1NM-PP1. Thiolutin treatment was performed as below. One microgram per milliliter of thiolutin (Sigma) or equivalent amount of DMSO was added to the cultures at 1 h after the addition of either auxin or solvent. Cells were then incubated for 30 min before sampling. To monitor the formation of Rad52 foci after phleomycin treatment, cells were cultured with 1 μg/ml of thiolutin or an equivalent amount of DMSO at 25 °C for 30 min and then further incubated with 10 μg/ml of phleomycin (InvivoGen) for 30 min. Cells were fixed with 70% ethanol for fluorescent microscopy imaging.

### Immunoblotting

Cell extracts were prepared following trichloroacetic acid (TCA) fixation. Cells were harvested and resuspended in 20% TCA. Cells were pelleted and then resuspended and broken with sodium dodecyl sulfate (SDS) containing sample buffer by glass beads breakage in a Multi Beads Shocker (Yasui Kikai). Samples were analyzed by SDS-PAGE followed by immunoblotting. Antibodies against the AID tag (Cosmo Bio, CAC-APC004AM) and the GFP tag (Torrey Pines Biolabs, TP401) and a mouse monoclonal antibody against α-tubulin (TAT1, Crick Cell Services) were used as primary antibodies.

### Fluorescent microscopy

For DNA volume quantification, cells were fixed with 70% ethanol and stained with 4′,6-diamidino-2-phenylindole (DAPI). A series of images were acquired along the *z*-axis (0.1 μm intervals) on a DeltaVision microscope system (Applied Precision). All images were deconvolved in SoftWoRx before counting voxels with a signal intensity over a relative threshold using the 3D object counter in Fiji [66]. The measurement of chromosomal distance and live cell tracking of chromatin locus were performed as previously described [17].

To calculate the nucleus to cytoplasm ratio (N/C ratio), we followed the idea of a previously published method [37]. The nuclear diameter, and cell length and width were measured from projections of LacI-GFP images using ImageJ. Nuclear and cellular volumes were calculated based on axial symmetry assuming simple geometries of nucleus (sphere) and cell (rod).

For Rad52 foci detection, a series of z-sectioned images were recorded without any fixation (Fig. 6b, c) or after cell fixation by 70% ethanol (Fig. 6d and Additional File 1: Fig. S8b) on the DeltaVision microscope. The z-sectioned images were then deconvolved and combined by the quick projection algorithm in SoftWoRx. Cells with one or more bright spots of Rad52 signal in the nucleus were counted as cells with Rad52 foci.

### Mean squared displacement calculation and Gaussian distribution fits

The MSD of individual tracks and the weighted mean of the MSDs were calculated using the @msdanalyzer Matlab class [67]. The mean squared displacement for a delay *τ* is calculated for individual tracks each of length *T*_*i*_, by finding the average of the squared displacement for all time separations *τ* along the track; this is an average over a total of *N*_*i*_(*τ*) ≈ (*T*_*i*_ − *τ*)/*δt* displacements, where *δt* = 0.02 s is the time resolution of the sampling. As is standard, to calculate a weighted mean MSD over several different tracks, each track for delay *τ* is weighted by *N*_*i*_(*τ*), since longer tracks will give a more reliable estimate:

$$ \left\langle m\left(\tau \right)\right\rangle =\frac{\sum_i{N}_i\left(\tau \right){m}_i\left(\tau \right)}{\sum_i{N}_i\left(\tau \right)}=\frac{1}{N\left(\tau \right)}{\sum}_i{N}_i\left(\tau \right){m}_i\left(\tau \right), $$

where *N*(*τ*) is the total number of displacements of delay *τ* over all tracks and *m*_*i*_(*τ*) is the MSD estimate for the *i*^th^ track. To calculate the standard error for the weighted mean MSD, the @msdanalyzer Matlab class uses a heuristic based on the weighted sample variance; however, instead we use the exact standard error given the weighting described, which can be shown by straightforward calculation to be

$$ {\sigma}_{\left\langle m\right\rangle}^2\left(\tau \right)=\frac{1}{N\left(\tau \right)}\left(\frac{1}{N\left(\tau \right)}{\sum}_i\left\{{N}_i\left(\tau \right)\left({m}_i^2\left(\tau \right)+{\sigma}_i^2\left(\tau \right)\right)\right\}-{\left\langle m\left(\tau \right)\right\rangle}^2\right). $$

The MSD of each track *m*_*i*_(*τ*) is fit for delays 0 ≤ *τ* ≤ 0.5 s to the power law function *aτ*^*α*^, first using a robust Nelder-Mead simplex algorithm (using matlab function “fminsearch”). The results of this first optimization are then used as input to the standard Matlab gradient-based optimization function “fitnlm.” For each track *i*, this produces an estimate of the power law exponent *α*_*i*_ ± *δα*_*i*_. A weighted histogram with weights in each track proportional to $1/\delta {\alpha}_i^2$ is calculated, normalized to give an estimate of the probability density of *α* (as shown by gray bars in the plots). We also estimate the weighted probability density of *α* using the kernel density smoothing method with Matlab function “ksdensity” with weighting $1/\delta {\alpha}_i^2$, and a Gaussian kernel. The kernel density estimate of *α* is then fit either using a single Gaussian

$$ p\left(\alpha \right)=\frac{1}{\sqrt{2\pi {\sigma}_{\alpha}^2}}\exp \left(-\frac{{\left(\alpha -\overline{\alpha}\right)}^2}{2{\sigma}_{\alpha}^2}\right), $$

or two Gaussians

$$ p\left(\alpha \right)=\frac{w}{\sqrt{2\pi {\sigma}_{\alpha 1}^2}}\exp \left(-\frac{{\left(\alpha -{\overline{\alpha}}_1\right)}^2}{2{\sigma}_{\alpha 1}^2}\right)+\frac{1-w}{\sqrt{2\pi {\sigma}_{\alpha 2}^2}}\exp \left(-\frac{{\left(\alpha -{\overline{\alpha}}_2\right)}^2}{2{\sigma}_{\alpha 2}^2}\right), $$

where the weight *w* is constrained such that 0 ≤ *w* ≤ 1.

### Monomer MSD for Rouse model with excluded volume

The subdiffusive motion of chromatin segments arises generically due to the fact that the chain is a connected object and must drag its local neighboring segments in order to diffuse. Using this intuition, a scaling argument can be developed to predict the monomer MSD for different polymer models.

For the ideal Rouse model, if a single monomer is to diffuse a mean square distance ⟨*r*^2^(*t*)⟩, then by the simple Rouse diffusive scaling law the number of monomers affected by this motion is determined by the relation ⟨*r*^2^(*t*)⟩ ∼ *n*(*t*); these *n* monomers must be dragged along for this displacement to occur. Each of these monomers moves within the local solvent environment, and so the effective friction is *ζ*(*t*) = *ζ*_0_*n*(*t*) = *ζ*_0_⟨*r*^2^(*t*)⟩, where *ζ*_0_ = 6*πηa* is the Stokes friction of a single monomer of size *a* in a solvent of viscosity *η*; *ζ*(*t*) determines the effective time-dependent diffusion constant of the monomer motion *D*(*t*) ∼ 1/*ζ*(*t*). The mean square displacement is then ⟨*r*^2^(*t*)⟩ ∼ *D*(*t*) × *t* = *t*/*ζ*(*t*) ∼ *t*/⟨*r*^2^(*t*)⟩ . Solving for ⟨*r*^2^(*t*)⟩, we have

$$ \left\langle {r}^2(t)\right\rangle \sim {t}^{1/2}. $$

However, real polymer chains cannot typically cross themselves due to excluded volume interactions and polymer theory predicts different scaling exponents *ν* for how the radius of gyration of the chain scales with length *N* (*R*_*g*_ ∼ *N*^*ν*^) dependent on the relative nature of chain-chain interactions to chain-solvent interactions [44]. The phantom Rouse chain gives an exponent *ν* = 1/2, whereas, for real chains in a “good” solvent, where attractive chain-chain interactions are not dominant compared to chain-solvent interactions, we expect *ν* > 1/2 and a more expanded chain. For a real chain in a good solvent with excluded volume, the same argument carries through: ⟨*r*^2^(*t*)⟩ ∼ *n*^2*ν*^, giving

$$ \left\langle {r}^2(t)\right\rangle \sim {t}^{\frac{2\nu }{1+2\nu }}. $$

Mean-field calculations for a polymer in good solvent give a scaling exponent *ν* = 3/5 [44] in 3 dimensions, while more sophisticated calculations give *ν* ≈ 0.588 [68]. Both values of *ν* give a prediction for MSD scaling exponent of an excluded volume Rouse polymer of *α* ≈ 0.54. This larger exponent for a Rouse model with excluded volume is due to the fact the chain is more expanded, meaning in order to diffuse a given distance, fewer monomers are involved, so there is less local friction and a larger MSD exponent.

### Simulation of a coarse-grained Rouse polymer with excluded volume

A chromosome arm of 3.6 Mb length is represented as a chain of 2436 beads, each with a radius of 25 nm. Based on previous simulations [69], one such bead is representative of 10 successive nucleosomes. Consecutive pairs of beads interact with each other in a linearly elastic manner. To model volume exclusion of the nucleosome chain, a simple constant repulsive force is applied to any two overlapping beads. Consequently, beads are not rigid bodies but allow ingression, as if a bead was a ten-nucleosome chain. Individual beads experience Brownian motion within a total spherical region of radius of 4.5 μm, reflecting little or no spatial constraint. The dynamical evolution of the chromosome chain is simulated by integrating the overdamped Langevin equation using a Euler integration method. The full mathematical description of the model is found in [70].

### Hi-C library preparation

Hi-C sample preparation was performed as described previously [17] with a slight modification to the proximity ligation step. To capture spatial proximity, chromatin was ligated at 25 °C for 4 h with T4 DNA ligase (New England BioLabs) without dilution. All other steps of the Hi-C sample preparation remained unchanged.

### Hi-C data analysis

Libraries for this project were sequenced using the Illumina HiSeq 4000 platform and 100-bp paired-end reads. Two biological replicate libraries were sequenced and the reads combined as single datasets with Juicer [71] version 1.5.6 using BWA [72] version 0.7.17 for alignment to genome version ASM294v2. Each biological replicate resulted in approximately 80 million read pairs. The replicate maps were subsequently merged using the Juicer mega.sh script into maps with approximately 160 million read pairs (Additional File 2: Table S2). Maps were further analyzed using R and strawR [73] or Hi-C Explorer [74, 75] version 3.2 (or 2.2 for format conversion).

TADs were identified using arrowhead in Juicer with default parameters or TopDom v0.0.2 [38] analysis of the Knight-Ruiz (KR)-normalized 2 kb resolution maps with the window.size = 10.

Contact probability decay curves were generated using the Hi-C Explorer hicPlotDistVsCounts function. Intra-arm bin pairs were selected from 2 kb resolution KR-normalized maps using strawR and converted to h5 format using Hi-C Explorer.

*Z*-scores were calculated from bin pairs using 2 kb resolution data normalized by the vc_sqrt method in Juicer. A *z*-score was calculated for intra-arm bin pairs that were separated by at least one bin; self-interacting and neighboring bin pairs were not considered. Genomic bands that included fewer than 15 bin pairs were also excluded from the analysis. For each genomic distance increment, the distribution of bin pair scores was used to calculate the *z*-score of bin pairs on the genomic separation band.

To calculate insulation scores, genome-wide KR-normalized interaction matrices were converted into 1D interaction vectors as described [17]. The 2 kb resolution maps were aggregated into 10 kb vectors using the mean interaction scores at 10–100 kb offsets in 10 kb increments. Interaction score profiles across either TopDom or arrowhead borders were calculated as the interaction score normalized by the mean score in a profile and log_2_ transformed. Profiles across borders were assembled into single profiles for each offset distance using the mean of the log_2_ normalized interaction score.

Aggregate Hi-C matrices around identified interactions were calculated using 2 kb resolution KR-normalized Hi-C matrices. Submatrices in 16 kb regions around interactions were extracted from genome-wide matrices using strawR [76]. Each submatrix was normalized by the mean score in the matrix, and submatrices were assembled into single profiles using the mean.

To prepare virtual 4C plots, the viewpoint profiles were extracted from the genome-wide 1 kb KR-normalized Hi-C matrix using strawR [76]. Interaction scores from the viewpoint within the plotted region were smoothed using Loess in geom_smooth() and ggplot2.

## Supplementary Information

**Additional file 1.** Supplementary Figures S1 – S8 with their legends and Table S1, yeast strains used in this study.

**Additional file 2.** Table S2, Hi-C library sequencing read statistics. (CSV 1 kb)

**Additional file 3.** Uncropped immunoblots.

**Additional file 4.** Review history.

## Data Availability

The sequencing datasets generated and analyzed in the current study are available in the Gene Expression Omnibus (GEO) database with accession number GSE143338, which can be found at https://www.ncbi.nlm.nih.gov/geo/query/acc.cgi?acc=GSE143338 [77]. The microscopy images analyzed in this study and raw data tables are available on Figshare and can be found at 10.6084/m9.figshare.12965306 [78].
