# Supplementary material for: Fission yeast condensin contributes to interphase chromatin organization and prevents transcription-coupled DNA damage
Source: Genome Biol. 2020 Nov 5;21:272. doi: 10.1186/s13059-020-02183-0 (PMC7643427; doi:10.1186/s13059-020-02183-0)
Supplement: Supplementary file 1 — Additional file 1. Supplementary Figures S1 – S8 with their legends and Table S1, yeast strains used in this study. [file 13059_2020_2183_MOESM1_ESM.docx]

**
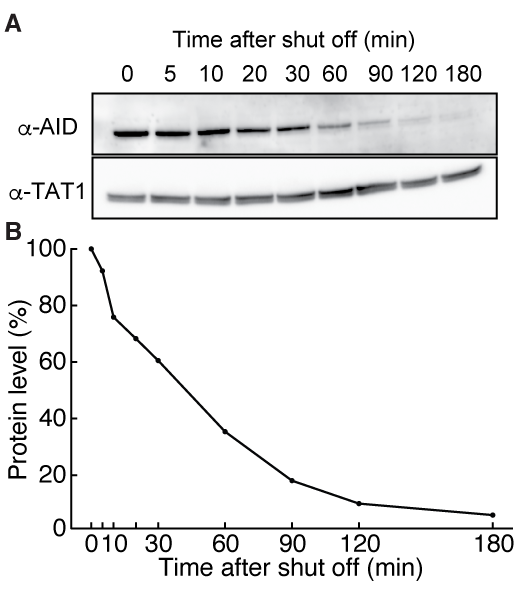
**

**Fig. S1** Time course of condensin depletion. **a** Western blot to confirm condensin depletion by promoter shut-off and auxin (NAA) addition to G2 arrested cells following *cdc2-as* inhibition by 1NM-PP1. Samples were taken at the indicated times and analyzed by western blotting. Cut14-AID was detected using an α-AID antibody. Tubulin (α-TAT1) served as a loading control. **b** Quantification of Cut14 protein levels on the blot shown in **a**. Signal intensities were normalized to tubulin.


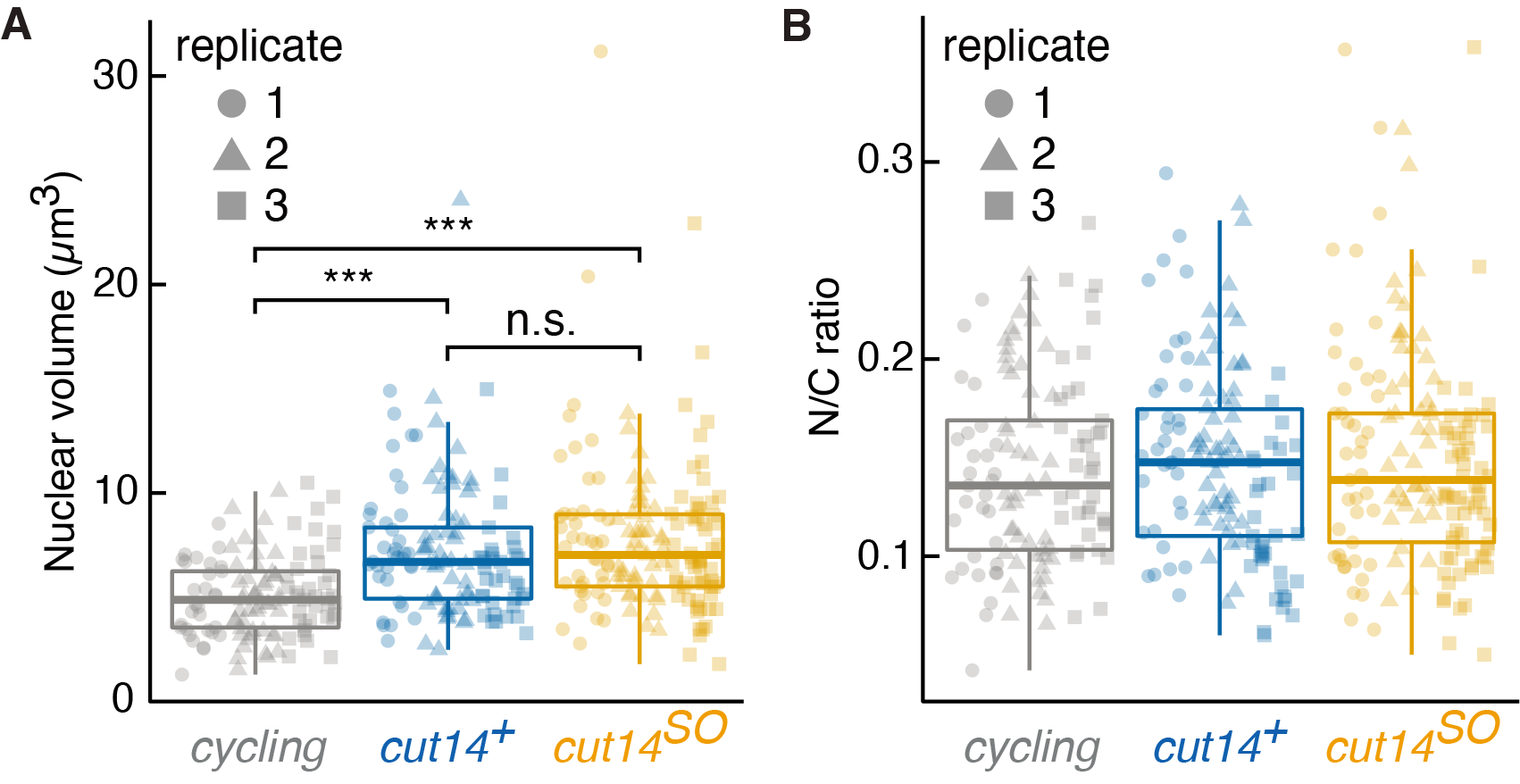


**Fig. S2** Condensin depletion does not impact on nuclear volume or the nucleus to cytoplasm (N/C) ratio. **a** Boxplot of nuclear volume in asynchronously growing (cycling) cells and of *cut14^+^* and *cut14^SO^* cells arrested in G2. Data from three biological replicates, represented by their distinct shapes (n > 30), is compiled in each boxplot. n.s.: no statistically significant difference, ***: p < 1.4 x 10^–7^. **b** Boxplot of the N/C ratio of the same cells in the indicated conditions. There was no statistically significant difference between the conditions.

**
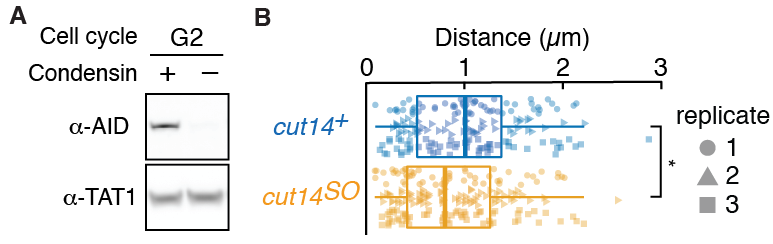
**

**Fig. S3** Reduced chromosomal distances in the absence of condensin. **a** Condensin depletion by promoter shutoff and NAA addition in G2 arrested cells following *cdc2-as* inhibition by 1NM-PP1 was confirmed by western blotting as in Fig. 1c. Cut14-AID was detected using an α-AID antibody. Tubulin (α-TAT1) served as a loading control. **b** Boxplot of chromosomal distances between the *lys1* locus and the 1.95 Mb point on chromosome I in *cut14^+^* control cells and following Cut14 depletion (*cut14^SO^*). Data from three biological replicates, identified by their distinct shapes, is compiled in one boxplots. The median distance between the loci was 1.01 µm in *cut14^+^* cells, which was reduced to 0.80 µm in *cut14^SO^* cells. *: *p* = 0.027.


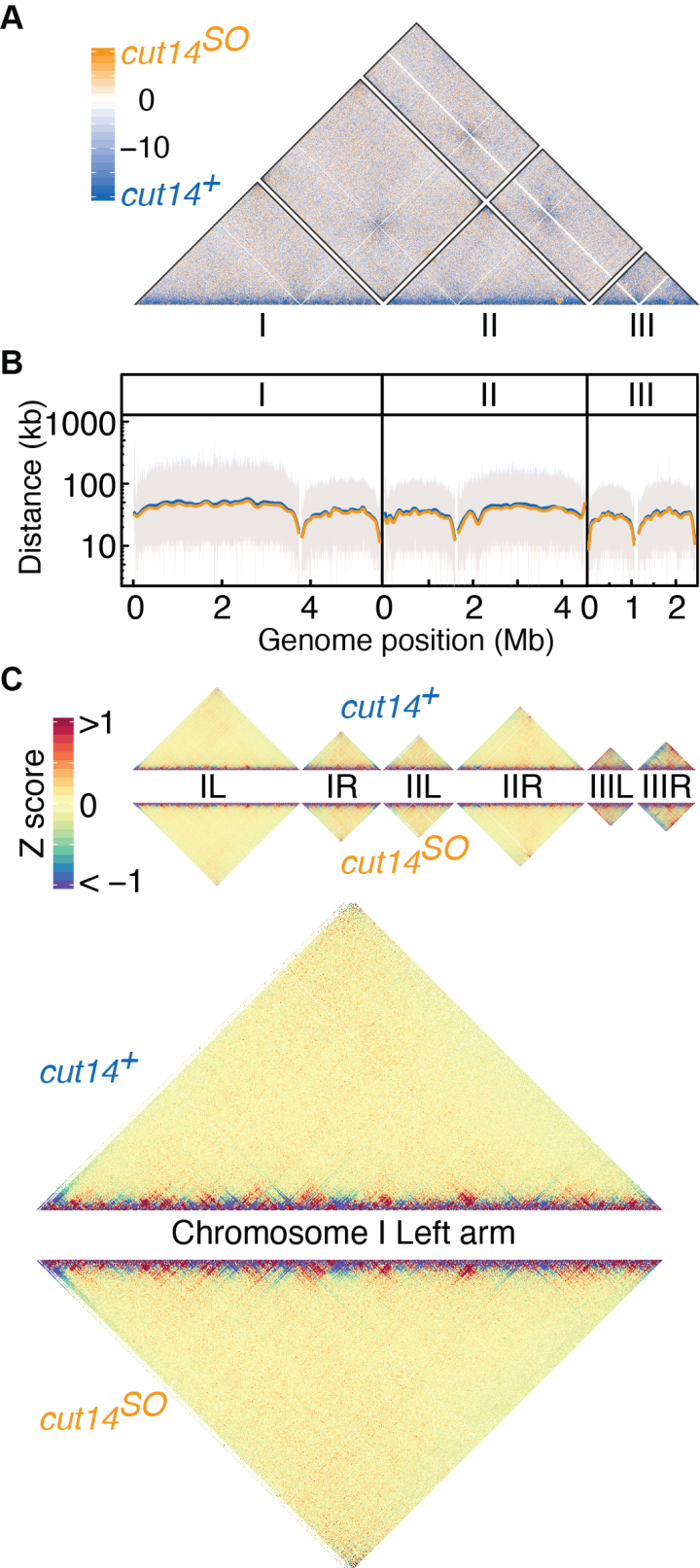


**Fig. S4** Additional Hi-C analyses. **a** Hi-C difference map of all three fission yeast chromosomes, comparing *cut14^+^* and *cut14^SO^* cells. **b** Median interacting distances as a function of chromosome coordinate. Medians (lines), as well as 25^th^ and 75^th^ percentiles (shadowed areas) of the interacting distances along chromosomes in *cut14^+^* control (blue) and *cut14^SO^* cells following condensin depletion (orange) are shown. Interacting distances are consistently shorter following condensin depletion. **c** Z-score Hi-C maps of the three fission yeast chromosomes in *cut14^+^* and *cut14^SO^* cells. A magnified view of the chromosome I left arm is shown.


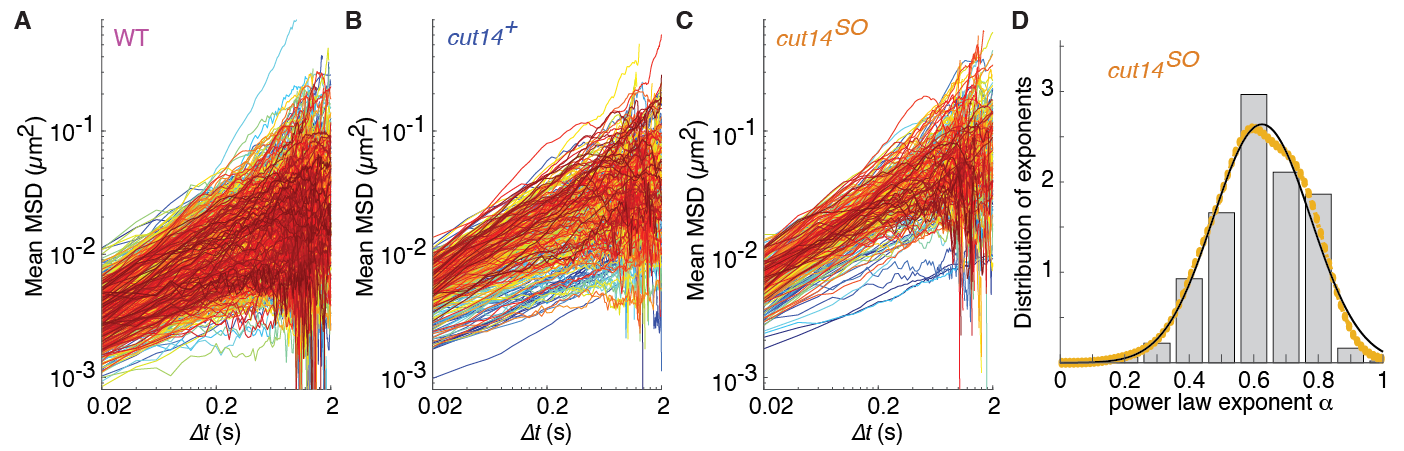


**Fig. S5** Additional MSD analyses. **a-c** Individual MSD trajectories in G2 arrested wild type (WT, **a**), *cut14^+^* control (*cut14^+^*, **b**) and condensin depleted (*cut14^SO^*, **c**) cells. **d** Histogram of MSD exponents in *cut14^SO^* cells, comparing a single Gaussian fit (black solid line) with a kernel density estimate of probability density of individual MSD exponents (yellow colored circles).


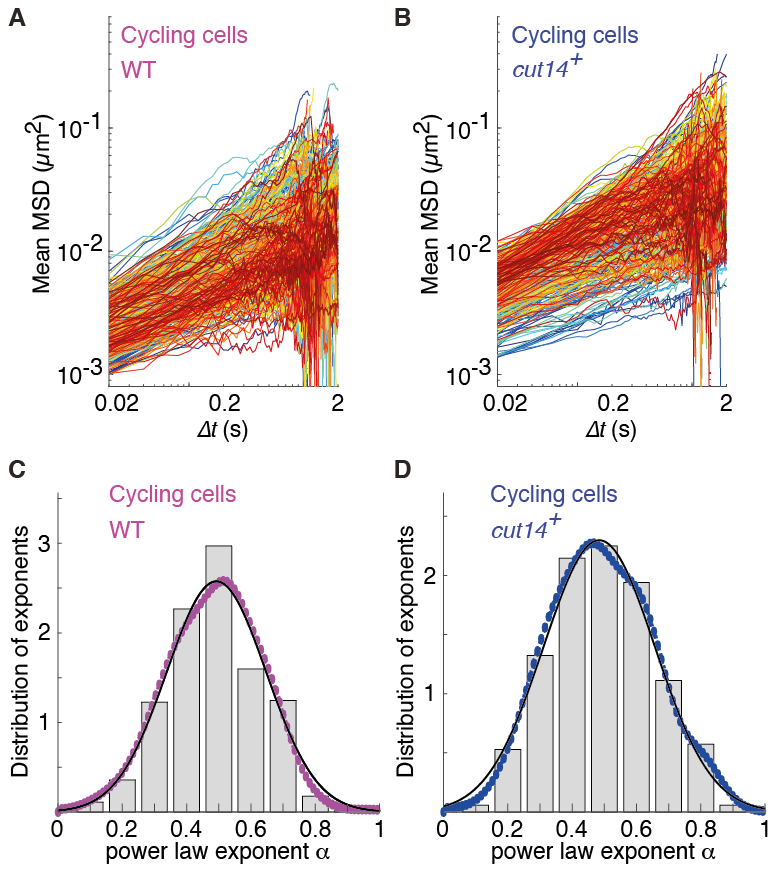


**Fig. S6** Chromatin motility analysis in unsynchronized cells. **a, b** Individual MSD trajectories in exponentially proliferating (cycling) wild type (WT, **a**) and *cut14^+^* control cells (*cut14^+^*, **b**) (n > 370). **c, d** Histograms of the MSD exponent distributions from **a** and **b**, displaying a single Gaussian fit (solid lines) and kernel density estimate of probability density of individual MSD exponents (colored circles).


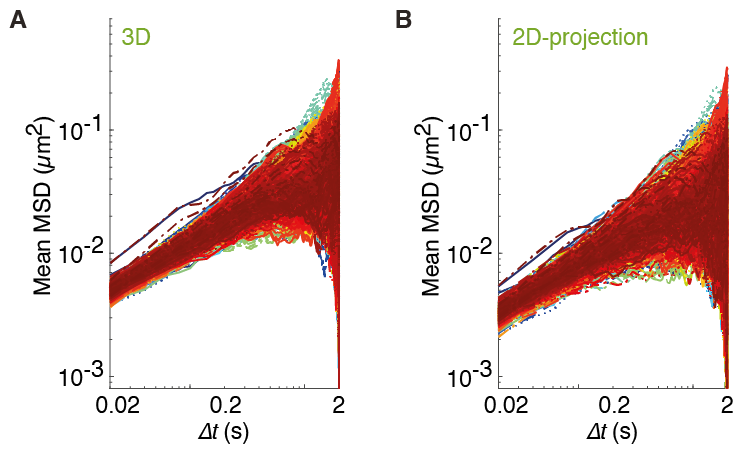


**Fig. S7** Simulation of a coarse-grained Rouse polymer with exclusion volume. **a, b** Individual MSD trajectories from the simulations in 3D (**a**), or following 2D projection (**b**).


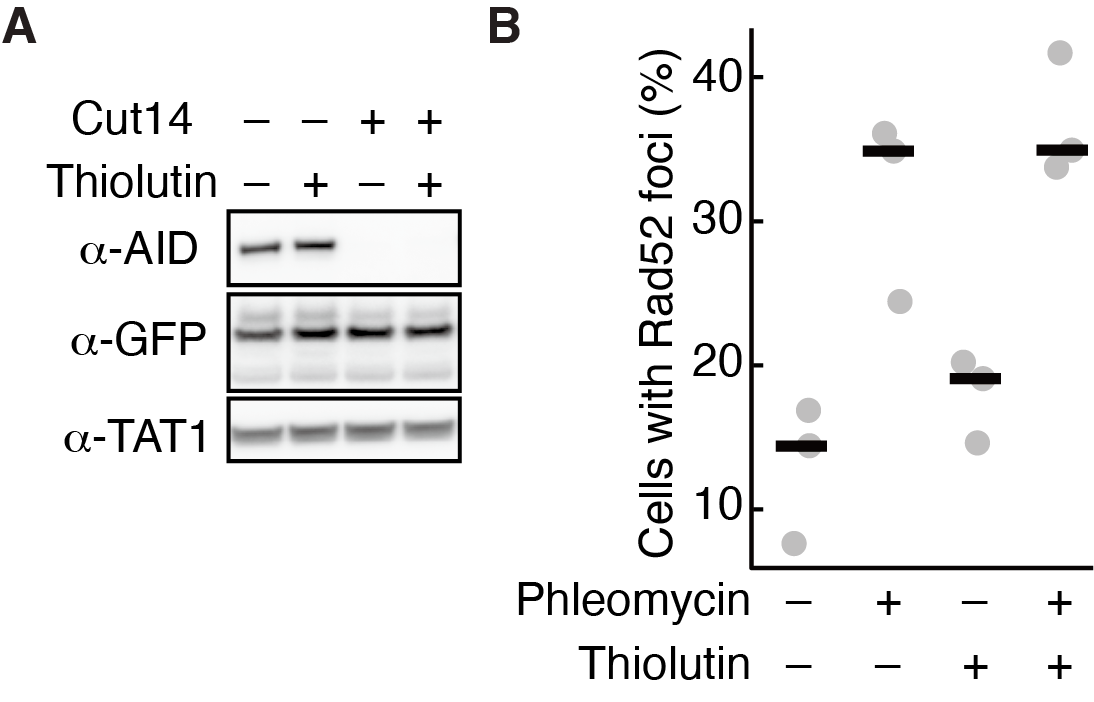


**Fig. S8** The DNA damage response remains intact following thiolutin treatment. **a** Rad52 protein levels remain stable following Cut14 depletion and thiolutin treatment. Western blot analysis is shown of Rad52-GFP protein levels following Cut14 depletion and thiolutin treatment. Control and *cut14^SO^* cells were treated with either 1 µg/ml thiolutin or solvent only for 30 minutes before sampling. Cut14-AID and Rad52-mVenus were detected by an α-AID antibody and an α-GFP antibody, respectively. Tubulin, detected by the α-TAT1 antibody, served as a loading control. **b** The phleomycin-induced DNA damage response is independent of transcription. The fraction of cells with Rad52 foci after the indicated treatments is shown. Dots and bars represent results from 3 biological replicates and their medians.

**Table S1: Strain list**

YUK404 *h^–^ nat-P_nmt81_-cut14-IAA-ura4^+^ ura4-D18* Fig.S1

*ade6::ade6^+^-P_adh15_-skp1-OsTIR1-kan^R^-P_adh15_-skp1-AtTIR1-2NLS*

YUK832 *h^+^ cdc2-as-M17-bsd aur1-P_nda3_-mCherry-atb2 nat-P_nmt81_-cut14-IAA-ura4^+^* Fig.1a,b,d,

*ade6::ade6^+^-P_adh15_-skp1-OsTIR1-kan^R^-P_adh15_-skp1-AtTIR1-2NLS*   2,3,S4

*his2 ura4-D18 leu1-32*

YUK842 *h^–^ cdc2-as-M17-bsd TetR-tdTom::leu1^+^ ChrII-3.6Mb::TetO-hphMX*  Fig.4,6a,

*LacI-eGFP::his7^+^ChrI-1.95Mb::LacO-natMX6 ura4-D18 leu1-32 lys1* S5,S6

*ade6::ade6^+^-P_adh15_-skp1-OsTIR1-kan^R^-P_adh15_-skp1-AtTIR1-2NLS*

YUK875 *h^–^ cdc2-as-M17-bsd aur1-P_nda3_-mCherry-atb2 nat-P_nmt81_-cut14-IAA-ura4^+^* Fig.1a-c,

*ade6::ade6^+^-P_adh15_-skp1-OsTIR1-kan^R^-P_adh15_-skp1-AtTIR1-2NLS* e,f,S2,S3

*Z-locus::natMX-TetR-tdTom ChrI-1.95Mb::hphMX-TetO*

*LacI-eGFP::his7^+^LacO::lys1^+^ ura4-D18 leu1-32*

YUK876 *h^–^ cdc2-as-M17-bsd aur1-P_nda3_-mCherry-atb2 nat-P_nmt81_-cut14-IAA-ura4^+^* Fig.4,6a,

*ade6::ade6^+^-P_adh15_-skp1-OsTIR1-kan^R^-P_adh15_-skp1-AtTIR1-2NLS* S5,S6

*TetR-tdTom::leu1^+^ ChrII-3.6Mb::TetO-hphMX*

*LacI-eGFP::his7^+^ChrI-1.95Mb::LacO-natMX6 ura4-D18 leu1-32 lys1*

YUK947  *h^+^ cdc2-as-M17-bsd aur1-P_nda3_-mCherry-atb2 nat-P_nmt81_-cut14-IAA-ura4^+^* Fig.6b-e

*ade6::ade6^+^-P_adh15_-skp1-OsTIR1-kan^R^-P_adh15_-skp1-AtTIR1-2NLS* S8

*rad52-mVenus-hph his2 ura4-D18 leu1-32*

YUK949  *h^–^ cdc2-as-M17-bsd aur1-P_nda3_-mCherry-atb2* Fig.6c

*ade6::ade6^+^-P_adh15_-skp1-OsTIR1-kan^R^-P_adh15_-skp1-AtTIR1-2NLS*

*rad52-mVenus-hph ura4-D18 leu1-32*
