# Supplementary material for: Fission yeast condensin contributes to interphase chromatin organization and prevents transcription-coupled DNA damage
Source: Genome Biol. 2020 Nov 5;21:272. doi: 10.1186/s13059-020-02183-0 (PMC7643427; doi:10.1186/s13059-020-02183-0)

Additional File 3: Uncropped immunoblots

Figure 1c

α-AID

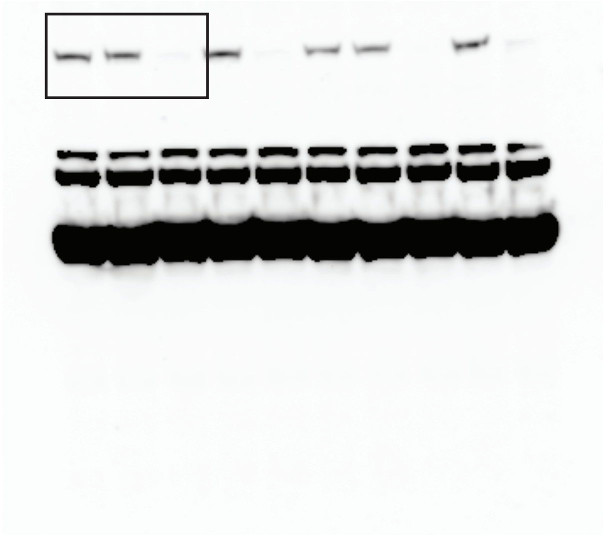

α-TAT1

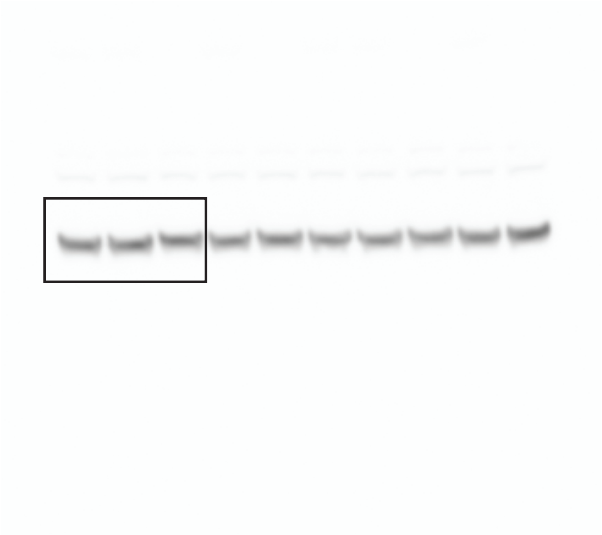

Figure S1a

α-AID

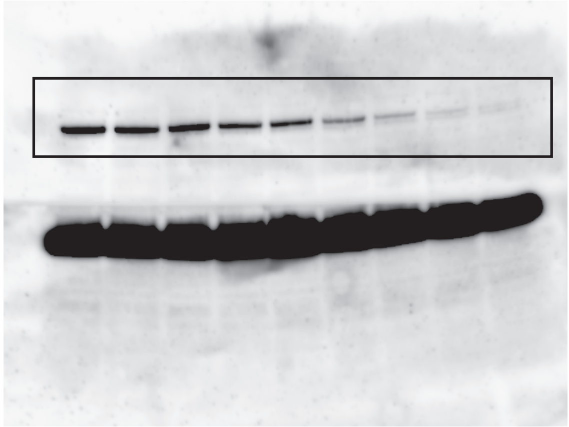

α-TAT1

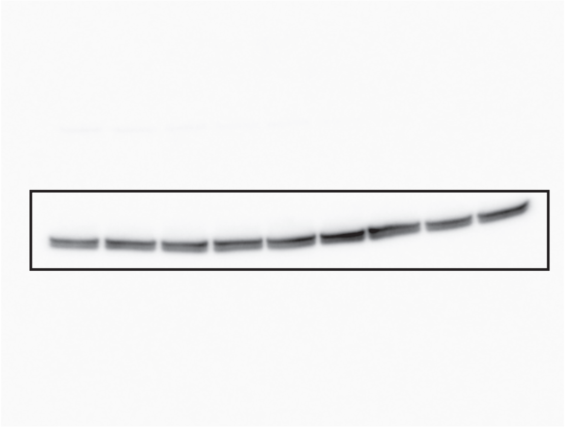

Figure S3a

α-AID

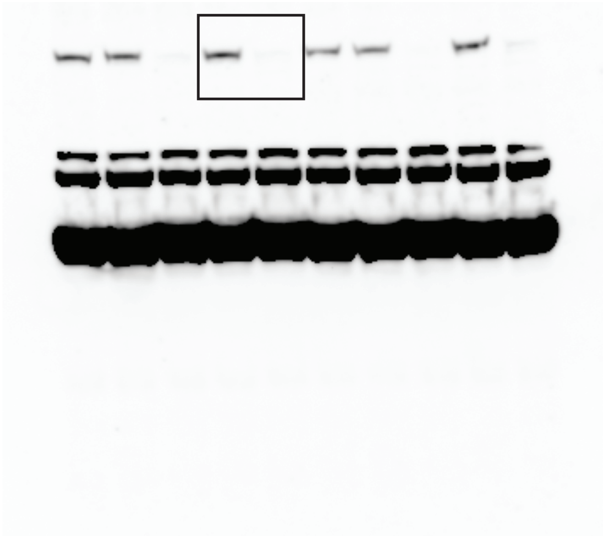

α-TAT1

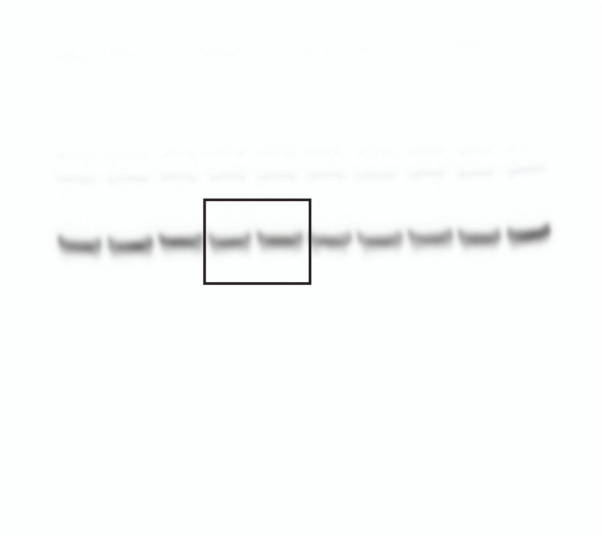

Figure S8a

α-AID α-GFP α-TAT1

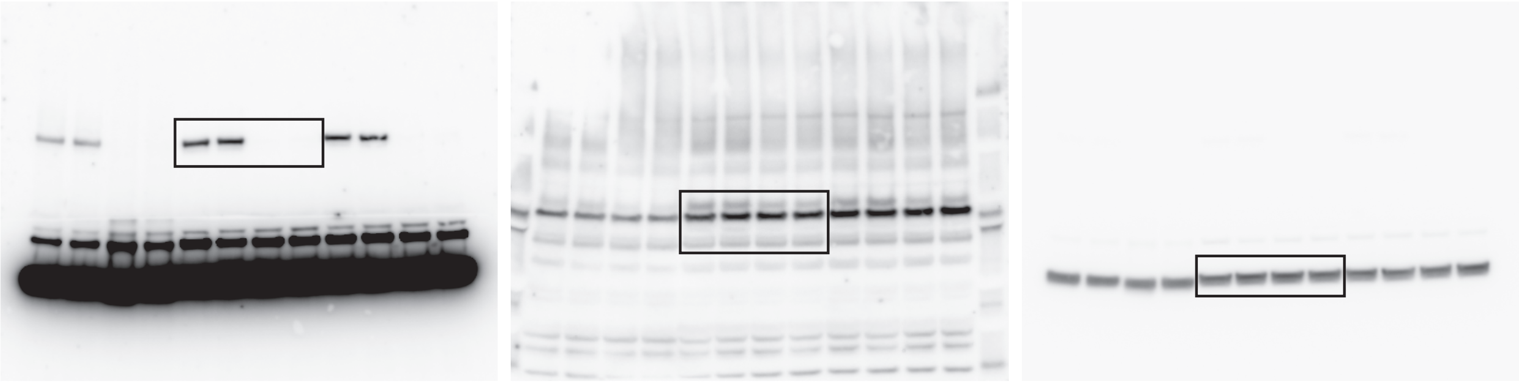

Supplement: Supplementary file 3 — Additional file 3. Uncropped immunoblots. [file 13059_2020_2183_MOESM3_ESM.pdf]
