# Supplementary material for: Fission yeast condensin contributes to interphase chromatin organization and prevents transcription-coupled DNA damage
Source: Genome Biol. 2020 Nov 5;21:272. doi: 10.1186/s13059-020-02183-0 (PMC7643427; doi:10.1186/s13059-020-02183-0)
Supplement: Supplementary file 4 — Additional file 4. Review history. [file 13059_2020_2183_MOESM4_ESM.docx]

**1^st^ round**

**Reviewer 1**

The manuscipt by Yasutaka Kakui and colleagues examines the role of fission yeast condensin in interphase nuclear architecture. For this, they use a system in with condensin subunit Cut14 can be efficiently depleted using a combination of promoter repression and acute degradation. In this manuscript, the authors suggest that condensin plays a role in maintaining chromatin architecture and controlling chromatin motility in interphase.They show that condensin depletion leads to an increase in DNA damage foci. From this they conclude that condensin chromatin organization has a role in protecting from DNA damage induction.

While some findings described in this paper are interesting to the field and may provide insights on the role of condensin in interphase, I cannot support the main conclusions as stated in the title: 'Fission yeast condensin maintains interphase chromatin organization to prevent spontaneous DNA damage'. Based on the data in this paper I would conclude that condensin does not maintain interphase chromatin organization, and has a role in DNA damage induction or DNA damage resolution. Based on analysis of Hi-C in presence vs absence of condensin, the authors draw strong conclusions based on very small differences that are unlikely to be biologically relevant. They also conclude that condensin-mediated chromatin organization prevents spontaneous DNA damage, which is not the only possible conclusion based on the data shown.

Specific points:

1. Overall, the authors often perform statistical analyses on genome-wide quantifications of the Hi-C data. As these are extremely large datasets, significant p-values are not informative. Take for example figure 3C. The authors here report a p-value of 3.7*10^-54, while I doubt that there is any biologically relevant difference. Based on the data shown in Figure 2 and 3, I would argue that the Hi-C is hardly different between cut14+ and cut14SO, leading to the conclusion that condensin is not required for maintenance of interphase genome organization.

2. In the Hi-C, the authors observe condensin dependent chromatin interactions that are qualitatively similar (but quantitively less prevalent) to interactions observed in mitosis. An alternative explanation for these findings could be that the Hi-C preparation is contaminated with mitotic cells. Could the authors show controls to verify that this is not the case?

3. The major point in figure 5 is that chromatin of condensin depleted nuclei mimics a beads-and-spring polymer with excluded volume. It will not be clear for anybody outside the modelling field why this is interesting. More explanation is needed to bring across the message of this figure.

4. The authors show that in fission yeast depleted of condensin, the Lc is increased. They then suggest that this is due to increased DNA damage. To strengthen this point, the authors should measure Lc in WT fission yeast with and without damage induction. If after damage the Lc has a similar value to the Lc of condensin depleted cells, this would support their model that DNA damage is the cause for the increase of Lc.

5. The authors use thiolutin as a transcription inhibitor and see a decrease in Rad52 foci formation in absence of condensin. From this they conclude that gene transcription potentially inflicts genome damage, and that condensin-mediated chromatin architecture normally prevents this damage. Inhibition of transcription can of course lead to many downstream effects. Therefore, it may very well be that not DNA damage induction is impaired after this treatment, but that simply the downstream signalling of these DNA breaks or other damage-inducing processes is affected. I suggest that the authors strengthen their statement by side by side inhibiting protein synthesis with an inhibitor of translation to then see whether Rad52 foci induction is not affected. Also, the authors should include a positive control in which DNA damage is still induced even in presence of Thiolutin, such as irradiation.

6. The increase in DNA damage foci may not only be consistent with the hypothesis that condensin is required to prevent the accumulation of spontaneous DNA damage, but might also indicate that DNA damage repair is less efficient. Could the authors comment on this?

Minor points

7. When discussing the auxin-inducible degron of Cut14, it would be good to reference the original paper in which this was first used (Nishimura, Nature Methods, 2009)

8. The authors claim the G2-arrest is successful because the cells grow longer than in cycling conditions. It would be good to show the controls of the cycling cells in figure 1a/b.

9. The authors conclude that domain architecture in fission yeast is dependent on cohesin rather than condensin following domain calling. However, instead of domain size I would argue that insulation score of the TADs is a more important measure of domain architecture. Could the authors include this analysis?

10. In figure 2e the authors only take into consideration interactions with a z-score greater than 2, while in figure 3d they only show Z-scores up to Z=1.

**Reviewer 2**

While the role of the structural-maintenance-of-chromosomes (SMC) complex condensin during mitosis is well known (i.e. compacting chromosomes by establishing long-range chromatin interactions), condensin function during interphase is less well understood. In this article, Kakui et al developed a system to study interphase chromatin organization in "condensin-free" fission yeast cells. Cells were arrested in G2 by inhibiting Cdk1 activity and subsequently depleted of Cut14 by using a degron system. Using these cells, the authors performed Hi-C and live-cell imaging to assess chromatin interactions and chromatin motility. With the collected experimental data as well as polymer simulations the authors reached the following conclusions among others:

1)	DNA volume is reduced upon loss of condensin.

2)	Condensin establishes long-range interactions in interphase.

3)	Condensin reduces chromatin movements in interphase.

4)	Loss of condensin induced DNA damage dependent on gene transcription.

Whereas the results showing that condensin reduces chromatin movement in interphase cells and that its loss results in DNA damage are interesting and the manuscript is well written, the conclusions drawn from Hi-C analyses are not convincing. The results presented might be suggestive but are not sufficient to support the authors conclusions. In its current form, the results seem more consistent with the conclusion that the overall interphase domain architecture is preserved in cells lacking condensin.

Specific comments:

(1) In Hi-C analyses, the differences between both samples are feeble and complex data processing was required to establish meaningful differences. For instance, when authors find a decrease in inter-chromosomes interaction (line 168; p:0.008), do the authors find significant differences for intra- and inter-arm interactions? Regardless of p-values, my point is about biological significance. In this sense, I would be more convinced by having examples of 4C-like plots using as baits regions that display robust differences.

(2) Previous studies have shown that the domain architecture in interphase S. pombe cells is mainly shaped by cohesion. It might be worth exploring whether loss of condensin in partial loss of function cohesin mutant cells causes additional changes in domain architecture. Moreover, cohesin-mediated organization shall be discussed in introduction and its relationship to current results should be considered in discussion.

(3) Lines 129-130: The authors use cell size as a proxy for nuclear size. It would be interesting to see whether the nuclear size is also decreased in cut14-SO cells. This is a very feasible experiment. The authors already have strains harboring nuclear fluorescent proteins such as lacI-GFP and tetR-Tomato which can be used to quantify nuclear volumes.

(4) Results showing that loss of condensin causes DNA damage is not totally unexpected since condensin mutants are highly sensitive to DNA damage agents (Aono et al., Nature 2002). Nevertheless, the connection with gene transcription is perhaps the most interesting observation of this work in terms of novelty. To further explore this topic, the authors can assess DNA volume, distance between lacO-tetO and chromatin motility tracking upon treatment with thiolutin. For instance, one would expect that the subpopulation with higher chromatin motility disappears when transcription is shut off.

(5) The authors draw contrast between their results and previous work. An important point that needs to considered is that condensin in this study is depleted using a degron system and the possibility remains that low levels of condesin remaining in cells is sufficient to promote chromosomal domain architecture (in any case, different exposure levels and time course might be needed to conclude that Cut14 is completely depleted).

**Authors’ response**

Reviewer 1 found that our ‘findings described in this paper are interesting to the field and may provide insights on the role of condensin in interphase’. At the same time, the reviewer queries the relatively small changes to chromatin architecture following condensin depletion in interphase.

1. We agree with the reviewer that the measurable impact of condensin on interphase chromatin architecture is small. In fact, one of the main conclusions from our study is indeed that (e.g. line 213) “the magnitude of condensin-mediated interactions is much less prominent in interphase” and that (line 194) “the fission yeast chromatin domain architecture in interphase is predominantly shaped by cohesin-mediated contacts”. Nevertheless, condensin affects interphase chromatin motility in a striking way and it plays a crucial role in protecting against transcription-induced DNA damage.

We have during the revision process performed additional analyses to better document the small but clearly discernible effects of condensin on interphase chromatin architecture. We extracted virtual 4C plots from the Hi-C data (a suggestion made by reviewer 2). These exemplify the increase of local chromatin contacts accompanied by a substantial reduction of long-range interactions following condensin depletion. An example is shown in the revised Figure 3a. Additionally, we present aggregate Hi-C matrices of condensin-dependent interactions (identified by their z-scores). These illustrate condensin-mediated loop formation in the interphase fission yeast nucleus. These additional analyses support a small but biologically relevant role of condensin in interphase genome organization.

2. A possible contamination of our interphase cultures with mitotic cells is a valid concern. Fortunately, chemical inhibition of cyclin-dependent kinase is a very efficient means to prevent mitotic entry and block cells in G2 phase. In the revised manuscript, we include additional controls to confirm the G2 arrest. This includes fluorescent microscopy observations, confirming that all cells arrest with a single nucleus and interphase microtubule arrays after 1NM-PP1 treatment.

In addition, G2-arrested cells, but not mitotic cells, grow longer during the arrest. In our experiments, the entire cell population shifts to a longer cell length, confirming the G2 arrest. These analyses are reported in the revised Figures 1a and b and the accompanying text. They confirm that no contaminant mitotic cells were detectable in our G2-arrested cultures.

3. It is widely believed that the often reported MSD exponent of 0.5 indicates that interphase chromatin behaves like an ideal Rouse polymer. We now reveal that this interpretation is misguided due to the fact that chromatin is not an ideal polymer, i.e. nucleosomes occupy a volume and cannot freely pass through each other. The simulation in our manuscript is a practical approach to establish the expected MSD of a realistic chromatin chain, which is greater than 0.5. This forms an integral part of our conclusion that condensin is responsible for constraining chromatin motility to its observed value of 0.5. This is better explained in the revised manuscript.

4. Our observations indeed suggest that chromatin motility and Lc increase in response to DNA damage. A direct causal relationship between DNA damage and an increased Lc has previously been demonstrated in budding yeast. We refer to and cite key publications from two different laboratories that document this. We agree with the reviewer that it will be important to perform similar experiments in fission yeast. This requires the establishment of an inducible DNA break system, of the sort used for the budding yeast studies. Unfortunately, given the complexity to establish such an inducible DNA break system in a new experimental organism, this approach goes beyond the scope of our current study.

5. The reviewer raises a critical point that our thiolutin experiment is insufficient to pinpoint transcription as the cause of DNA damage following condensin depletion. In response, we analyzed the alternative scenario in which transcription is required for Rad52 foci formation as part of the DNA damage response. To do this, we analyzed Rad52 foci formation following treatment with the gamma irradiation mimetic phleomycin. Pretreatment of the cells with thiolutin did not interfere with Rad52 foci formation in response to phleomycin. Therefore, thiolutin does not interfere with the response to exogenous DNA damage. This adds weight to the interpretation that transcription produces endogenous DNA damage following condensin depletion.

As an aside, we note that cellular Rad52 protein levels remain unaltered following thiolutin treatment. These new results are contained in an additional Figure S8.

6. The reviewer alludes to the seminal observation by Aono et al. 2002, which revealed a role for condensin in the repair of UV-induced DNA damage. We referred to this publication in the introduction and have during the revision added an additional mention and reference to condensin’s role during DNA repair to our discussion.

7. We thank the reviewer for pointing out this omission. A reference to the original paper that describes the use of an auxin-inducible degron has been added where we describe our combined promoter shut-off and auxin-inducible degron approach.

8. This is related to point 2, above. A comparison of the lengths of cells grown in cycling conditions and following G2 arrest is now contained in our revised Figure panels 1a and 1b.

9. The reviewer rightly suggests that insulation score analysis is an important aspect when considering chromatin domain boundaries. In response, we have analyzed insulation at domain boundaries in the presence and absence of condensin and found that insulation remained unaffected. These results are presented in new Figure panels 2e and f.

10. Apologies for this misrepresentation. Figure 3d, which is now part of Figure S4c, shows all interactions at any z-score. To visualize regions of enriched interactions, a z-score of greater than one is depicted red in this diagram. For the new Figure panels 3c and d, we select a strong subset of these interactions, those with a z-score greater than 2, for further analysis. This is now better explained.

Reviewer 2 finds that our results are ‘interesting and the manuscript well written’, but also probes our conclusions from the Hi-C experiment and makes a number of suggestions for how to improve this analysis.

1. We agree with the reviewer that the measurable impact of condensin on interphase chromatin architecture is small. In fact, one of the main conclusions from our study is indeed that (e.g. line 213) “the magnitude of condensin-mediated interactions is much less prominent in interphase” and (line 194) “that the fission yeast chromatin domain architecture in interphase is predominantly shaped by cohesin-mediated contacts”. Nevertheless, condensin affects interphase chromatin motility in a striking way and it plays a crucial role in protecting against transcription-induced DNA damage.

We have during the revision process performed additional analyses to better document the small but clearly discernible effects of condensin on interphase chromatin architecture. As suggested by the reviewer, we extracted virtual 4C plots from the Hi-C data. These exemplify the increase of local chromatin contacts, accompanied by a substantial reduction of long-range interactions following condensin depletion. An example is shown in the revised Figure 3a.

The Hi-C analysis revealed that interaction changes in the intra-arm, inter-arm and inter-chromosome categories were all significant (p = 0.03, 0.03, 0.008, respectively).

Additionally, we show aggregate Hi-C matrices of condensin-dependent interactions (identified by their z-scores). The aggregate matrices illustrate condensin-mediated loop formation in the interphase fission yeast nucleus. These additional analyses suggest a small but biologically relevant role of condensin in interphase genome organization.

2. To study the relationship between condensin and cohesin in controlling chromatin architecture will be an important next project. The required genetic tools to independently and efficiently deplete both condensin and cohesin, as well as the expected complexity of the resultant datasets, mean that this is a considerable project in its own right that goes beyond the scope of our current study. Instead, we now clearly describe cohesin-mediated domain organization in our introduction and use it as a reference point in our results and discussion sections.

3. As the reviewer suggested, we have measured nuclear size using the LacI-GFP signal. As expected from the cell size increase during the G2 arrest, the nuclear volume also increased when compared to cycling cells. Importantly, the increase was similar in both cut14+ and cut14SO cells. The nuclear volume was unaffected by condensin depletion and the nucleus to cytoplasm ratio remained constant in all cells. These measurements are documented in a new Figure S2.

4. The reviewer asks us to further explore the consequences of transcription-induced DNA damage by characterizing the effect of transcription inhibition by thiolutin. As suggested by the reviewer, we have measured the impact of transcription-induced DNA damage on DNA volume. Thiolutin did not affect the DNA volume in cut14+ cells, however thiolutin suppressed the DNA volume reduction observed in cut14SO cells. These results support the conclusion that condensin protects interphase chromatin architecture against transcription-induced DNA damage. They further suggest a link between DNA damage and DNA volume compaction, which we discuss. These new results are contained in an additional Figure panel 6e.

5. In response to the reviewer’s very pertinent question about the condensin depletion efficiency, we have quantified Cut14 levels over time following promoter shut-off and auxin-inducible degron activation. This is shown in a new Figure S1 and confirms that condensin levels are close to undetectable after three hours, the time when our experiments are performed. Despite the dramatic reduction of condensin levels, interphase domain architecture remained unaffected. The latter conclusion is now substantiated by an insulation score analysis (a suggestion from reviewer 1) that is contained in new Figure panels 2e and f.

**2^nd^ round**

**Reviewer 1**

The authors have satisfactory addressed most of my comments. I have two remaining points that can both be addressed with textual changes.

1) The modeling in figure 5 will still not be clear to most readers. The explanation the authors offered in their ‘response to the reviewers’ was very clarifying. I would recommend that the authors also include such an explanation in the actual manuscript.

2) This point is more important. The authors have now convinced me that condensin does play a role in the interphase genome, but the effects are clearly very minor. I would therefore urge the authors to tone down the title to better reflect their findings.

Once the authors have addressed these two points, I would say that this paper is highly suitable for publication in Genome Biology.

**Reviewer 2**

The authors have satisfactorily addressed most of my concerns. Indeed, the suppression by Thiolutin displayed in revised Fig. 6e is remarkable as well as the data for nuclear size and condensin depletion (revised Figs. S1 and S2). Regarding the measurable impact of condensin on interphase chromatin architecture I would like to see more examples like that shown in revised Fig. 3a. Otherwise, I would like to see a modified abstract acknowledging that “the magnitude of condensin-mediated interactions is much less prominent in interphase” and “that the fission yeast chromatin domain architecture in interphase is predominantly shaped by cohesin-mediated contacts”.
